# Identification of potential quality markers in Indonesia’s Arabica specialty coffee using GC/MS-based metabolomics approach

**DOI:** 10.1007/s11306-023-02051-5

**Published:** 2023-10-25

**Authors:** Fitri Amalia, Tomoya Irifune, Tetsuji Takegami, Yusianto, Ucu Sumirat, Sastia Prama Putri, Eiichiro Fukusaki

**Affiliations:** 1 Department of Biotechnology, Graduate School of Engineering, Osaka University, 2-1 Yamadaoka, Suita, Osaka 565-0871 Japan; 2 Indonesian Coffee and Cocoa Research Institute, Jl. PB. Sudirman 90, Jember, East Java 68118 Indonesia; 3 Industrial Biotechnology Division, Institute for Open and Transdisciplinary Research Initiatives, Osaka University, 2-1 Yamadaoka, Suita, Osaka 565-0871 Japan; 4 Osaka University Shimadzu Omics Innovation Research Laboratories, 2-1 Yamadaoka, Suita, Osaka 565-0871 Japan

**Keywords:** Specialty, Coffee, Arabica, Metabolomics, GC/MS, OPLS

## Abstract

**Introduction:**

The cupping test is a widely used method for quality assessment of Arabica coffee. However, the cupping test is limited by the low number of certified panelists and the low throughput. Therefore, an analytical-based quality assessment may be a promising tool to complement the cupping test. A present, there is no report investigating quality marker candidates, focusing only on “specialty” grade Arabica coffee from Indonesia.

**Objective:**

This study identified the potential quality marker(s) in Arabica Specialty coffee at different stages (green beans, roasted beans, and brewed coffee.

**Methods:**

The metabolite profiles of ten different Arabica specialty-grade coffees were analyzed with different cup scores using gas chromatography–mass spectrometry (GC/MS). From the ten samples, green coffee beans, roasted coffee beans, and brewed coffee were selected. In addition, an orthogonal projection to latent structure (OPLS) regression analysis was conducted to obtain a potential quality marker based on the variable importance in projection (VIP). The potential quality marker(s) were validated by GC/MS metabolome profiling and OPLS analysis of different sets of samples consisting of 35 Arabica specialty-grade coffee samples.

**Results:**

In Arabica coffee samples, the OPLS model of the three stages showed galactinol to have a high VIP score. Galactinol showed a consistent positive correlation with cup scores at all stages of coffee production (green beans, roasted beans, and brewed coffee). The correlation suggests galactinol is a potential quality marker after further validation using different samples.

**Conclusion:**

GC/MS combined with OPLS regression analysis suggested galactinol as a quality marker and provide an early screening method for Arabica coffee quality that complements the cupping test performed by certified panelists.

**Supplementary Information:**

The online version contains supplementary material available at 10.1007/s11306-023-02051-5.

## Introduction

High-quality coffee, such as specialty coffee, has recently gained considerable attention (International Trade Centre, 2012). Therefore, the importance of quality assessment of high-quality coffee is also increasing. *Coffea arabica* (Arabica) and *Coffea canephora* (Robusta) are commercially essential coffee species. Arabica coffee is considered as a high-quality coffee due to its original superior sensory properties to that of Robusta coffee (Putri & Fukusaki, 2018). Therefore, the quality assessment of high-quality Arabica coffee is critical.

The standardized quality assessment method for Arabica coffee is the cupping test established by the Specialty Coffee Association of America (SCAA). Based on this standard, the specialty coffee grade is given to the coffee with a final score higher than 80 out of 100 and later influences the price of the Arabica coffee in the market (Specialty Coffee Association of America, 2015; Tolessa et al., 2017). Although the SCAA cupping test is considered the most reliable method for evaluating Arabica coffee quality, the test is limited by a lack of skilled panelists worldwide and low throughput due to the decreasing sensitivity of panelists during evaluation. Analytical-based quality assessment has been demonstrated to avoid these limitations as the coffee flavor is strongly related to the presence of compounds (Farah et al., 2006; Ribeiro et al., 2012). Identifying marker compounds correlated with cupping quality may be a promising way to complement cupping test-based quality assessment. Hence, a high-throughput quality assessment can be achieved by providing rapid screening methods using marker compounds of coffee quality.

Food metabolomics is the application of metabolomics technique on a complex food matrix, obtaining comprehensive profile of food metabolome and correlating it with various factors that could influence food quality and food safety (Wu et al., 2022). The metabolomics techniques also have been employed in coffee studies to investigate the coffee quality and its correlated compounds to identify marker for coffee quality determination (Iwasa et al., 2015; Toci & Farah, 2014). However, these studies targeted various quality coffee with a wide range of final cup scores, including “specialty” and “not specialty” grades, while there are more classes within the specialty grade itself based on the SCAA protocol, namely “very good” (80–84.99), “excellent” (85–89.99) and “outstanding” (90–100) (Specialty Coffee Association of America, 2015). Furthermore, previous studies investigating the metabolites correlated with coffee quality covered different classes of compounds with different analytical instruments, such as LC/MS and HS-GC/MS, in brewed coffee and powdered roasted coffee (Rocchetti et al., 2020; Sittipod et al., 2019, 2020). However, no reports are available on investigating metabolites correlated with coffee quality using only “specialty” grade coffee that could be applied to all coffee forms (green coffee beans, roasted coffee beans, and brewed coffee). Hence, a metabolomic approach was applied to investigate the metabolites correlated with coffee quality, described as cup score, using specialty coffee Arabica from Indonesia. Metabolic profiling of aqueous extracts of green beans, roasted beans, and brewed coffee was subjected to gas chromatography–mass spectrometry (GC/MS) analysis. Subsequently, a prediction model of cupping scores was created using orthogonal projection to latent structure (OPLS) regression analysis. Based on the variable importance in projection (VIP), an indicator of the importance of compounds involved in constructing the model, the compounds highly correlated with coffee quality were suggested. The study is the first to analyze the correlation between metabolites and coffee quality to identify candidate marker compounds for quality determination using green beans, roasted beans, and brewed specialty Arabica.

## Materials and methods

### Chemicals

The chemicals used in this study are described in previous studies (Jumhawan et al., 2013; Putri et al., 2019). All the chemicals were of analytical grade. Methanol (99.8%), pyridine (99.5%), and ribitol were purchased from FUJIFILM Wako Pure Chemical Industries, Ltd. (Osaka, Japan). Chloroform (99%) was purchased from Kishida Chemicals (Osaka, Japan). Methoxyamine hydrochloride was purchased from Sigma-Aldrich (Milwaukee, WI, USA). *N*-Methyl-*N*-(trimethylsilyl) trifluoroacetamide (MSTFA) and a standard alkane mixture (C10–C40) were purchased from GL Science Inc. (Tokyo, Japan).

### Coffee beans sample collection

All coffee beans were collected and provided by the Indonesian Coffee and Cocoa Research Institute (ICCRI), Jember, Indonesia, during the Indonesian Specialty Coffee Competition (Kontes Kopi Spesialti Indonesia). All the samples were coded in number (Table S1) and divided into two sample sets. The sample set 1 consisted of ten kinds of green (raw, not roasted) and roasted Arabica coffee beans, coded as A/008, A/034, A/009, A/001, A/018, A/026, A/067, A/025, A/063, and A/039, were used for constructing the model (Table S1—Sample set 1). Furthermore, the sample set 2 consisted thirty-five green Arabica coffee beans, were used to validate the findings from the model constructed from the sample set 1 by expanding the number of samples (Table S1—Sample set 2). All roasted beans were roasted under uniform conditions at the ICCRI following the SCAA cup protocol. The roasting conditions were as follows: coffee beans were roasted in a Probat-Werke von Gimborn Maschinenfabrik GmbH model BRZ 2 (Probat, Rhein, Germany) at 205 °C for 10 min until the coffee bean developed brown color measured according to Agtron “Gourmet” 63.0 and then were air-cooled for 5 min. The ten kinds of roasted and green beans were stored at − 30 °C until metabolite profiling.

### Sensory analysis

The sensory analysis was conducted at the ICCRI for all the samples. The sensory analysis was performed within 24 h after the roasting process according to the standard protocol published by the SCAA for certified panelists (Q-Grader). Ten parameters were rated from 0 to 10, with 0 indicating the least and ten indicating the strongest taste. The total cup score was obtained by accumulating the scores for all parameters and determining the coffee grade (Specialty Coffee Association of America, 2015).

### Metabolite extraction for green and roasted coffee beans

Extraction was performed following the protocol described previously with some modifications. Coffee beans (green and roasted) were taken in a 50 mL tube (Yasui Kikai Co.) with a metal cone (Yasui Kikai Co.) and quenched using liquid nitrogen. The coffee beans were ground at 2000 rpm for 30 s using a multi-bead shocker (Yasui Kikai Co.), and the step was repeated three times to produce a fine coffee powder. Powdered coffee beans (15 mg) were transferred into a 2 mL tube, and three replicates were prepared (*n* = 3) for each sample. A blank was prepared, and all the steps were followed together with other samples. Hydrophilic low-molecular-weight compounds in powdered coffee beans were extracted using 1 mL of single-phase solvent mixture of methanol, ultrapure water, and chloroform at a ratio of 2.5/1/1 (v/v/v) containing ribitol (12 µg/mL) as an internal standard. The samples were incubated at 37 °C and 1200 rpm for 30 min and centrifuged at 4 °C and 16,000×*g* for 3 min. The supernatant (900 mL) was transferred into a 1.5 mL tube, and 400 mL of ultrapure water was added to the tube. The mixture was then vortexed and centrifuged at 4 °C and 16,000×*g* for 3 min. The aqueous phase (200 µL) was transferred into a new 1.5 mL tube with a pierced cap. The solvent was evaporated using vacuum centrifugation for 50 min, followed by overnight lyophilization (Amalia et al., 2021).

### Coffee brew sample preparation

Coffee brew samples were prepared from ten kinds of roasted Arabica coffee beans, coded as A/008, A/034, A/009, A/001, A/018, A/026, A/067, A/025, A/063, and A/039 (Table S1—Sample set 1). Coffee brew preparation, including weighing, grinding, and brewing, was performed in technical replicates (*n* = 3). Each sample weighed approximately 4 g and was ground using a BISTRO electric coffee grinder (BODUM; Trigen, Switzerland). Approximately 3.5 g of coffee powder was taken in a ceramic cup, and 75 µL ribitol solution (12 mg/mL) was added as an internal standard. Hot water (temperature 93 °C) was added to the cup. The coffee brew was allowed to sit for 3 min and 30 s. Then, 10 mL of coffee brew was transferred to a 15 mL centrifuge tube. A blank was prepared using only hot water and an internal standard (ribitol 12 mg/mL).

The coffee brew (1.5 mL) was transferred to a 2 mL tube and centrifuged at 16,000×*g* for 10 min at 4 °C. The supernatant and blank (20 µL) were transferred to a 1.5 mL tube with a pierced cap. For quality control (QC) purposes, 20 µL of supernatant from the samples was collected in one pool by transferring it into a 2 mL tube. The sample extracts collected in the QC pool were homogenized, and 20 µL of the extract was transferred into a new 1.5 mL centrifuge tube with a pierced cap. The QC and blank samples were lyophilized overnight.

### Metabolite derivatization

The derivatization process consists of two steps, oximation and trimethylsilylation. The lyophilized green and roasted bean extracts and coffee brew were subjected to oximation by adding 100 µL methoxyamine hydrochloride (20 mg/mL in pyridine) and then vortexed and incubated at 1200 rpm and 30 °C for 90 min, respectively. Subsequently, 50 µL MSTFA was added, and the mixture was vortexed and incubated at 1200 rpm and 37 °C for 30 min, respectively. The sample mixture (100 µL) was transferred into a vial before the GC/MS analysis (Amalia et al., 2021).

### GC/MS analysis

The GC/MS analysis protocol has been described previously, with some modifications. The samples were subjected to GC/MS analysis immediately after the derivatization. GC/MS analysis was performed using GCMSQP2010 Ultra (Shimadzu, Kyoto, Japan) for green and roasted bean extracts and GCMS-TQ8030 (Shimadzu, Kyoto, Japan) for the coffee brew. The instruments were equipped with a 30 m × 0.25 mm i.d. fused silica capillary column coated with 0.25 µm InertCap 5MS/NP (GL Science, Inc., Tokyo, Japan) and an AOC-20i/s (Shimadzu, Kyoto, Japan) as an autosampler. System control and data acquisition were conducted using GCMS solution software (Shimadzu, Kyoto, Japan). The mass spectrometer was tuned and calibrated before the analysis. A standard alkane mixture (C10–C40) was injected at the beginning of the retention index calculation. The derivatized green and roasted bean extract samples (1 µL) were injected in split mode (12:1(v/v)) at an injection temperature of 230 °C. The derivatized coffee brew samples (1 µL) were injected in split mode (25:1(v/v)) at an injection temperature of 270 °C. All samples were injected randomly with QC injections (1 μL) every four or five injections. Helium was used as the carrier gas at a flow rate of 1.12 mL/min and linear velocity of 39 cm/s. The column temperature was held at 80 °C for 2 min, increased by 15 °C/min to 330 °C, and finally kept at 330 °C for 6 min. The transfer line temperature was 250 °C. The ion source temperatures were 200 °C and 280 °C for GCMSQP2010 Ultra and GCMS-TQ8030, respectively. The ions were generated via electron ionization (EI) at 70 eV. EI mass spectra were recorded at 6.67 scans per second for GCMSQP2010 Ultra and 20 scans per second for GCMS-TQ8030 over the mass range of m/z 85–500 (Amalia et al., 2021).

### Data processing

In this study, the data processing procedure was carried out with several modifications. The chromatogram data were converted to the netCDF format using the GCMS Solution software package (Shimadzu, Kyoto, Japan). The netCDF files were then converted into the ABF format using the Reifycs Abf Converter (https://www.reifycs.com/AbfConverter/). The ABF file format was then used for peak detection, deconvolution analysis, identification, and alignment using MS-DIAL version 4.18 (Lai et al., 2018; Tsugawa et al., 2015, 2019), a freely available software (http://prime.psc.riken.jp/Metabolomics_Software/MS-DIAL/). The peak detection parameters were set with a minimum height of 1000 amplitude and a linear weight moving average smoothing method. The smoothing level was set to five scans, and the average peak width was 20 scans. The σ-window value was set to 0.5, and the EI spectra cut-off was set to 200 amplitudes for the deconvolution analysis. The retention index of the alkane mixture (C10–C40) and the GL-Science DB Library (InertCap 5MS-NP, Kovats RI, 494 records; http://prime.psc.riken.jp/Metabolomics_Software/MS-DIAL/GCMS%20DB_InertCap%205MS-NP_GLscience.msp) were used for peak identification. The parameters for peak identification and alignment were set as follows: retention index (RI) tolerance of 10, retention time (RT) tolerance of 0.5 min, *m/z* tolerance of 0.5 Da, EI similarity cut-off of 70%, and identification score of 70%. Metabolite annotation was conducted by comparing the mass spectra and RI with those of the GL-Science DB library. After peak detection and annotation, the heights of the detected peaks were normalized to those of the internal standard peaks. The data matrix consisted of the raw and normalized heights of the peaks. After creating the data matrix, metabolite filtering was conducted to exclude peaks that were not of biological origin by referring to the blank. Moreover, peaks with a relative standard deviation of more than 30% in QC and a raw height of less than 10,000 (green and roasted coffee beans) and 5000 (coffee brew) were removed. A final data matrix with filtered and assigned peaks was constructed and used for further analysis (Amalia et al., 2021).

### Multivariate analysis

The relative intensity data of the annotated compounds were used as explanatory variables, and the final cup score data were used as response variables. OPLS regression analysis was performed using commercially available SIMCA-P+ version 13.0.3 (Umetrics, Umeå, Sweden). The data matrix was subjected to principal component analysis (PCA) and OPLS with auto-scale and without transformation to equally analyze all the obtained metabolites (van den Berg et al., 2006). The constructed OPLS model also evaluated using cross-validation ANOVA (CV-ANOVA) analysis (Eriksson et al., 2008).

### Quantitation of marker candidate

The quality marker candidate was quantified in the sample using the calibration curve of the galactinol standard prepared at various concentrations. The concentrations of the standards were 0.02, 0.08, 0.2, 0.8, 1.6, 2.4, 3.2 and 4 µg/mL in ultrapure water. The sample extracts were prepared from green coffee beans. The standard solution and the sample extracts were lyophilized overnight. After derivatization, the standard and extract of the samples were analyzed using GC/MS with the same parameters as described previously, except that the ion monitoring mode was set at *m/z* 204. After data processing, the standard calibration curve was plotted, and sample quantitation and Student *t*-test were performed using Microsoft Excel.

## Results and discussion

### Metabolic profiling of Arabica specialty coffee

GC/MS-based metabolic profiling was performed on aqueous extracts of green and roasted coffee beans and brewed coffee of ten kinds of specialty Arabica coffee, and found 124 peaks detected in green coffee beans, 279 in roasted coffee beans, and 134 in brewed coffee. In green coffee beans, there were 68 annotated metabolites, whereas, in roasted coffee beans, there were 72 annotated metabolites. In brewed coffee, there were 63 annotated metabolites, whereas the rest were unknown. The annotated metabolites in the three different sample types were classified as amino acids, sugars, sugar alcohols, organic acids, and other compounds (Tables S2, S3, S4). Roasted coffee bean extract has the highest number of metabolites, as various reactions occur during roasting processes, such as pyrolysis, the Maillard reaction, and carbohydrate caramelization (Clifford et al., 2018). These reactions can transform naturally occurring compounds in green coffee beans into various compounds that contribute to the overall aroma of coffee (Buffo, 2018; Clifford et al., 2018). Nevertheless, GC/MS-based metabolite profiling offers more comprehensive coverage compared to similar biochemical composition studies, which described more targeted metabolites such as caffeine, sucrose, *N*-methylnicotinic acid, and chlorogenic acid (Farah et al., 2006; Perrone et al., 2008). Therefore, numerous metabolites could be used as explanatory variables in multivariate analysis to investigate the coffee quality in different samples to corroborate previous studies. The relative intensity data of the detected metabolites were used for multivariate analysis. PCA was performed to examine the data structure before the final cup score model construction by OPLS regression analysis. The data showed good reproducibility since the sample replicates from the same class and the quality control replicates were clustered together (Figs. S1, S2, S3). The PCA score plot of green beans (Fig. S1) showed four different clusters. The samples coded as A/008, A/001, A/018, A/009, and A/026 clustered together, while A/034 and A/063 clustered at center of axis. The same result also showed that the sample coded as A/067 and A/39 clustered together, and A/025 was separated from the rest of the samples. In the PCA score plot of roasted beans (Fig. S2), A/008 and A/034 were clustered together at the left axis of PC1, while A/026 and A/025 at the right axis of PC1. The sample coded as A/001, A/018, A/009, A/067, and A/039 at the middle of the axis of the roasted beans’ PCA score plot. The PCA score plot of brewed coffee (Fig. S3) showed two different clusters where A/025 were separated from the rest of samples in which clustered together. Although each sample stage has different clustering dynamics, the PCA result of green coffee beans, roasted coffee beans, and brewed coffee showed similar pattern where A/008 which has the highest cup score was separated and located at opposite axis to A/025 which has relatively lower cup score. Further, the data were used for OPLS regression analysis to construct a model between metabolite profile and cupping score.

### Selection of quality marker candidates for Arabica specialty coffee

**Fig. 1:**
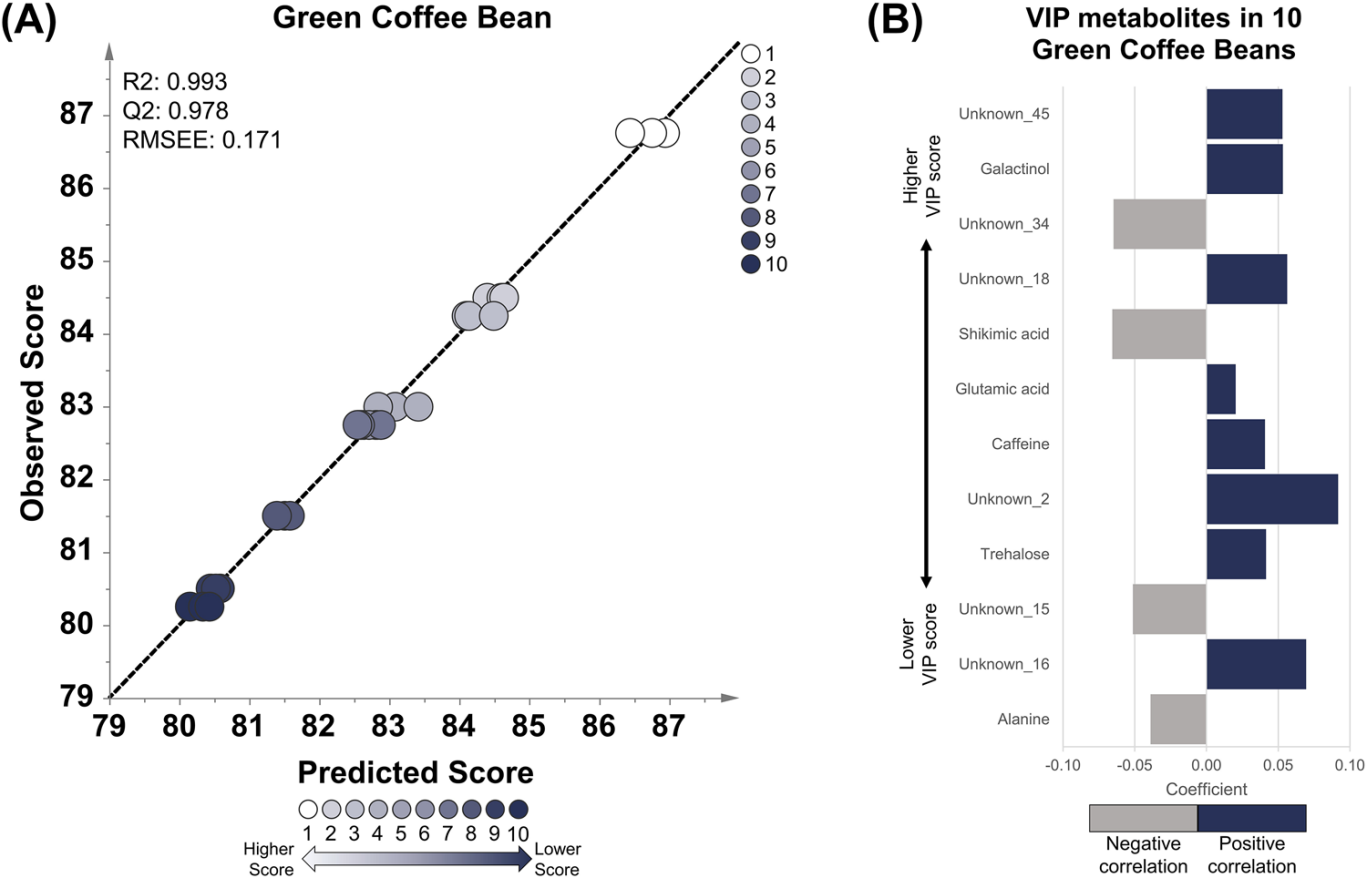
OPLS regression model using green coffee beans of 10 specialty coffee with a score range of 80.25–86.75. **A** The OPLS model shows good linearity and robustness based on the R2, Q2, and RMSEE values. **B** The important metabolites with a VIP value of more than 1.5. Twelve important metabolites showing positive (blue) and negative correlation (gray). Eight metabolites positively correlated with cup score, including unknown_45, galactinol, unknown_18, glutamic acid, caffeine, unknown_2, trehalose, and unknown_16. Four metabolites with negative correlation are unknown_34, shikimic acid, unknown_15, and alanine

**Fig. 2:**
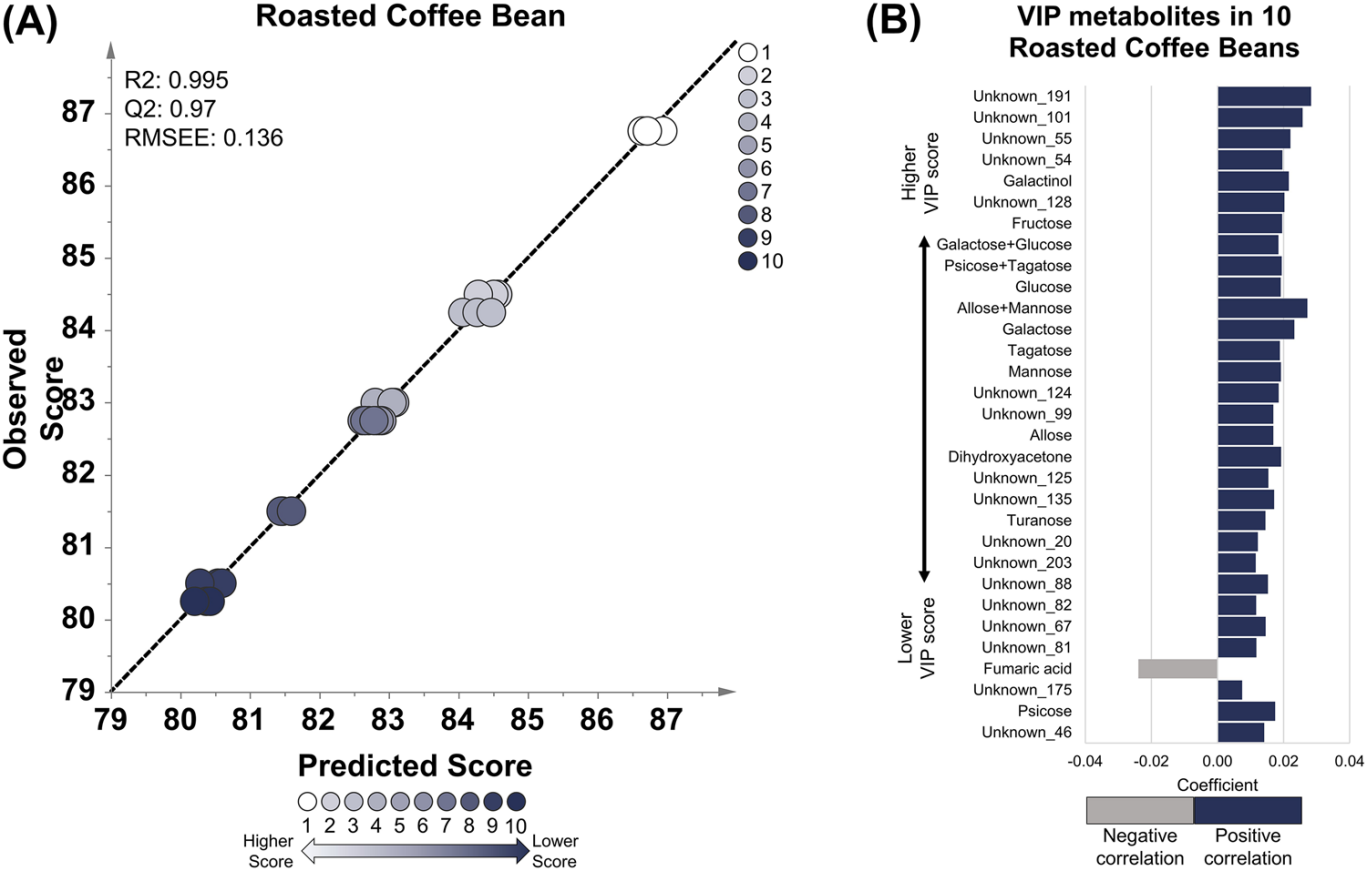
OPLS regression model using roasted coffee beans of 10 specialty coffee with a score range of 80.25–86.75. **A** The OPLS model shows good linearity and robustness based on the R2, Q2, and RMSEE values. **B** The important metabolites with a VIP value of more than 1.5. Thirty-one important metabolites showed positive (blue) and negative correlations (gray). Most sugar compounds correlate positively with cup scores, while fumaric acid correlates negatively

**Fig. 3:**
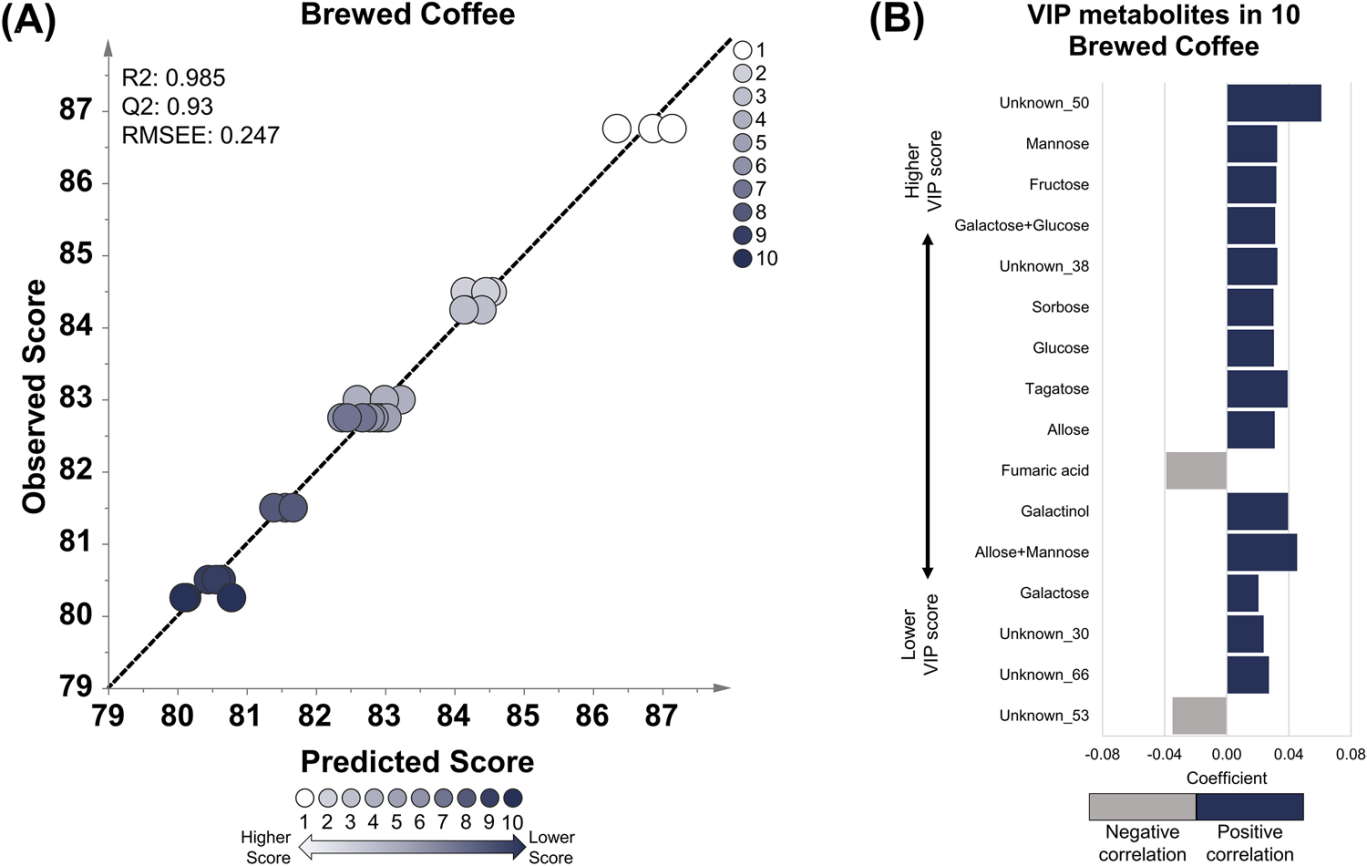
OPLS regression model using brewed coffee of 10 specialty coffee with a score range of 80.25–86.75. **A** The OPLS model shows good linearity and robustness based on the R2, Q2, and RMSEE values. **B** The important metabolites with a VIP value of more than 1.5. Sixteen important metabolites showed positive (blue) and negative correlations (gray). Most sugar compounds correlate positively with cup scores. Fumaric acid and unknown_53 correlates negatively with the cup score

OPLS regression analysis was performed to create a final cup score model with the final score of the cupping test as a response variable and the metabolites detected in green coffee beans, roasted coffee beans, and brewed coffee as explanatory variables (Figs. 1, 2, 3). The OPLS multivariate method was used to provide better interpretability than partial least squares (PLS) analysis by removing variation from X that is not correlated to Y (Trygg & Wold, 2002). Moreover, the OPLS method has been applied in food metabolomics research to investigate important metabolites correlated with Japanese sake, soy sauce, and civet coffee. Based on these studies, the OPLS method could find the metabolites that could predict the quality of sake, soy sauce, and marker to differentiate civet coffee from regular coffee (Jumhawan et al., 2016; Mimura et al., 2014; Yamamoto et al., 2014). Hence, the same OPLS method was used to explore the metabolites that could predict the coffee quality as well. Figures 1A, 2A, and 3A showed the OPLS regression results for green, roasted, and brewed coffee, respectively. All the OPLS models had R2 and Q2 values greater than 0.9, indicating that the model had a good correlation between the final score and the explanatory metabolites of each stage of the coffee sample (green beans, roasted beans, and brewed) (Alexander et al., 2015; Eriksson et al., 2006; Varmuza & Filzmoser, 2009; Worley & Powers, 2013). Furthermore, the OPLS-R model evaluation using CV-ANOVA, showed that the constructed model also significant as shown in low *p*-value (Table S5) (Eriksson et al., 2008).

The metabolites correlated with the cup score model in each coffee sample were sorted based on their VIP values (Tables S6, S7, S8). VIP is a parameter that shows the cumulative measure of the influence of individual metabolites on a model (Galindo-Prieto et al., 2014). Metabolites with VIP values greater than 1 are considered important for the model (Ikram et al., 2020; Mabuchi et al., 2019). In this study, metabolites with VIP values of more than 1.5 in different stages of coffee samples (green beans, roasted beans, and brewed) were selected as potential markers for coffee quality. Twelve metabolites in the green coffee beans were correlated with cup scores, consisting of annotated and unknown metabolites with positive or negative correlations (Fig. 1B). Galactinol, glutamic acid, caffeine, trehalose, and unknown compound number 2, 16, 18, and 45 were the metabolites that exhibited a positive correlation with the cup score. Meanwhile, shikimic acid, alanine, and unknown compounds 15 and 34 were negatively correlated with the cup score. Thirty-one metabolites in roasted beans correlated with the cup score, and more than half of the list were unknown metabolites (Fig. 2B). The unknown metabolites, coded as unknown number 191, 101, 55, 54, 128, 124, 99, 125, 135, 20, 203, 88, 82, 67, 81, 175, and 46, have positive correlation with coffee cup scores in roasted bean samples. Various sugar compounds such as fructose, glucose, galactose, tagatose, mannose, allose, turanose, galactose + glucose, psicose + tagatose, and allose + mannose were found to be positively correlated with coffee cup scores. The sugar alcohol galactinol and another compound, dihydroxyacetone, were also found to be positively correlated. Fumaric acid was the only compound with a negative correlation with the cup score in roasted coffee beans. Sixteen metabolites were highly correlated with cup scores in brewed coffee (Fig. 3B). Similar to roasted beans, several sugar compounds, such as fructose, glucose, galactose, tagatose, mannose, allose, sorbose, galactose + glucose, and allose + mannose, along with galactinol, were positively correlated with cup scores. Unknown metabolites coded as unknown number 50, 38, 30, and 66 also have positive correlation with cup scores. Fumaric acid was also found to be negatively correlated with the unknown compound number 53. Based on this result, numerous compounds from various classes, such as amino acids, organic acids, sugar alcohols, and sugar compounds, correlate with coffee quality. Coffee-related compounds, such as caffeine, trigonelline (*N*-methylnicotinic acid), sucrose, and chlorogenic acid, were also detected. However, only caffeine is present in one of the three OPLS models (green coffee beans). Therefore, the metabolites found in this study may complement previous coffee quality studies that targeted coffee-related compounds (Farah et al., 2006; Perrone et al., 2008).

Caffeine is a heat-stable alkaloid responsible for 10% of the bitter taste of coffee beverages (Buffo, 2018). In this study, caffeine was positively correlated with coffee quality in the green coffee bean model. Caffeine is often used to evaluate coffee quality, and several studies have investigated the correlation between caffeine content and cup quality (Belay et al., 2008; Farah et al., 2006; Franca et al., 2005). Previous studies have found a positive correlation between caffeine and high-quality green coffee beans, which is consistent with the results of this study. In addition, a previous study found that caffeine, to a lesser extent than trigonelline and 3,4-dicaffeoilquinic acid, had a positive correlation with coffee cup quality in green beans (Farah et al., 2006).

Glutamic acid and alanine are amino acids that correlated with coffee quality in green coffee beans. Glutamic acid and alanine are the main amino acids in green coffee beans and possibly influence coffee quality because amino acids are flavor precursors for flavor development during roasting (Buffo, 2018; Hu et al., 2001). During the roasting process, amino acids degrade and form volatile compounds, which determine the coffee color and antioxidant activity (Buffo, 2018; Poisson et al., 2009; Yu et al., 2013). Numerous studies have been conducted on how glutamic acid and alanine contribute to the formation of volatile compounds, such as pyrazines, in the Maillard reaction (Poisson et al., 2009; Yu et al., 2013). However, it remains unclear why opposite trend was observed between glutamic acid and alanine for coffee quality. Therefore, further studies are necessary to explain the role of amino acids in the quality of green coffee beans.

Organic acids, namely shikimic acid in green coffee beans and fumaric acid in roasted coffee beans and brewed coffee, were negatively correlated with coffee quality in this study. This is the first correlation report between shikimic acid content and coffee quality. Fumaric acid is an aliphatic acid naturally present in coffee beans and can be formed by the degradation of malic acid during the roasting process (Farah & De Lima, 2019; Jham et al., 2002). To the best of our knowledge, no previous reports have described the correlation between fumaric and coffee quality. Organic acids in coffee beans play various roles in coffee acidity and flavor. Organic acids could also be desirable or undesirable depending on their predominance in coffee (Farah & De Lima, 2019). Several organic acids have been characterized in terms of flavor and acidity intensity (Farah & De Lima, 2019; Jham et al., 2002). However, fumaric acid and shikimic acid have not yet been characterized, and further investigations related to these acids are required.

Carbohydrates are a major component of green coffee beans and make up 50–60% of their dry weight (Arya & Rao, 2007; Buffo, 2018; Farah, 2012). Carbohydrates are also precursors for the Maillard reaction and contribute to the organoleptic appeal of roasted coffee and coffee beverages, such as color, aroma, acidity, and viscosity (Arya & Rao, 2007; Buffo, 2018; Farah, 2012; Redgewell & Fischer, 2006). Moreover, carbohydrates influence the sweetness of coffee beverages (Sunarharum et al., 2014). Hence, carbohydrates and sugar derivatives are important constituents that correlate with coffee quality. In this study, trehalose and galactinol levels were positively correlated with coffee quality in green coffee beans. Trehalose and galactinol previously detected in coffee showed a relation with geographical origin or postharvest process (Amalia et al., 2021; da Silva Taveira et al., 2014; Miao et al., 2022; Putri et al., 2019). However, no study has discussed the relationship between these metabolites and coffee quality. In roasted beans and brewed coffee, various monosaccharides, disaccharides, and galactinol positively correlate with the coffee quality. Sugar compounds provide coffee beverages with sweet taste perceptions (Seninde & Chambers, 2020). The sugar compounds found in roasted beans might have resulted from the degradation of carbohydrates in green coffee beans through pyrolysis and caramelization during the roasting process (Buffo, 2018; Cordoba et al., 2019). Since sugar compounds are soluble in water, they could be detected further in brewed coffee and influence the taste of coffee beverages (Cordoba et al., 2019; Flament, 2001).

**Fig. 4:**
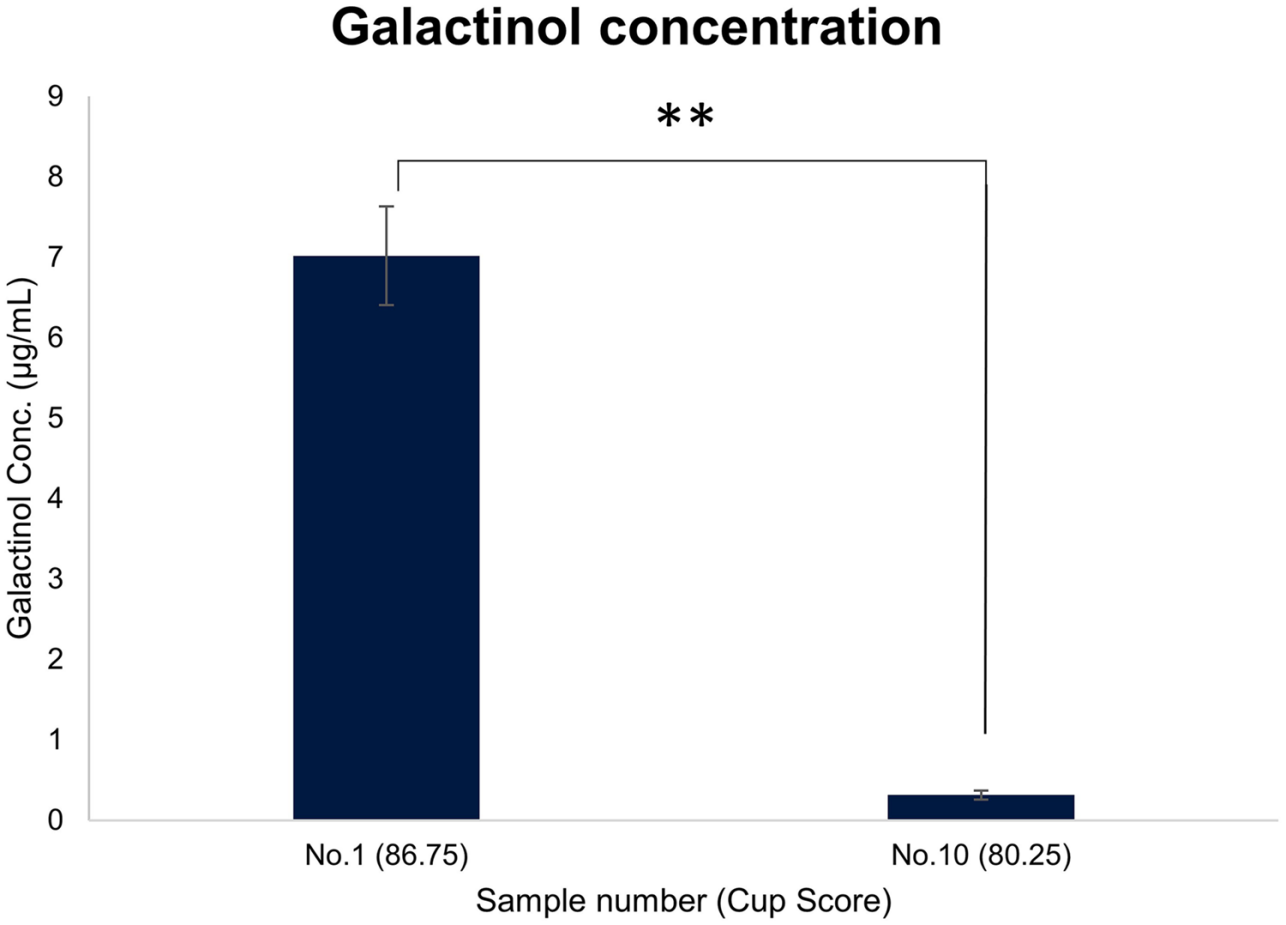
Quantification of galactinol concentration. The quantification of galactinol concentration in the green coffee bean sample with a high cup score and the sample with a lower cup score. The concentration difference is statistically significant according to the Student *t*-test method with a *p*-value < 0.01

Among all the selected metabolites in the models of different stages of coffee samples, galactinol consistently correlated positively with the final coffee cup score. Thus, further analysis was conducted to investigate the correlation of galactinol relative intensity with seven different taste attributes at different stages of coffee samples using the Pearson correlation coefficient (Table 1). The results showed galactinol had a significant correlation (*p*-value = 0.05) with several taste attributes in the cupping test of all stages of coffee samples. In green coffee beans, galactinol was highly correlated with flavor, aftertaste, acidity, and overall attributes. Galactinol was also highly correlated with fragrance/aroma, flavor, aftertaste, acidity, and overall attributes in both roasted beans and brewed samples. The finding was also supported by an OPLS regression analysis of these attributes using different stages of coffee samples, in which galactinol had a VIP value of more than 1.5 (data not shown). Furthermore, the galactinol quantification was performed by constructing the calibration curve of galactinol standard (Fig. S4). The intensity of galactinol detected was 3653–805,643 unit in the range of 0.02–4 μg of galactinol. The calibration curve showed good correlation with R2 = 0.99 and produced the formula that correlates intensity and the amount of galactinol. Then the formula is used to calculate the galactinol amount in the sample divided by the volume of green coffee beans extract before spin dry (200 μL). Based on the calibration curve formula, the galactinol content in the green coffee bean samples with the highest and lowest scores could be obtained, 7.02 μg/mL and 0.31 μg/mL respectively (Fig. 4). The results showed that the highest score samples had 22 times higher galactinol content than the lowest score samples.

**Table 1:** Pearson correlation analysis of galactinol content and the coffee taste attributes in different stages of coffee samples

Sample	Fragrance/aroma	Flavor	Aftertaste	Acidity	Body	Balance	Overall	Final score
Green bean	0.582	0.914*	0.955*	0.77*	0.395	− 0.219	0.704*	0.754*
Roasted bean	0.806*	0.916*	0.882*	0.868*	0.523	− 0.247	0.796*	0.831*
Brewed coffee	0.766*	0.972*	0.947*	0.909*	0.415	− 0.422	0.839*	0.718*

*p-value < 0.05

### Applicability of galactinol for quality determination of specialty Arabica green bean

**Fig. 5:**
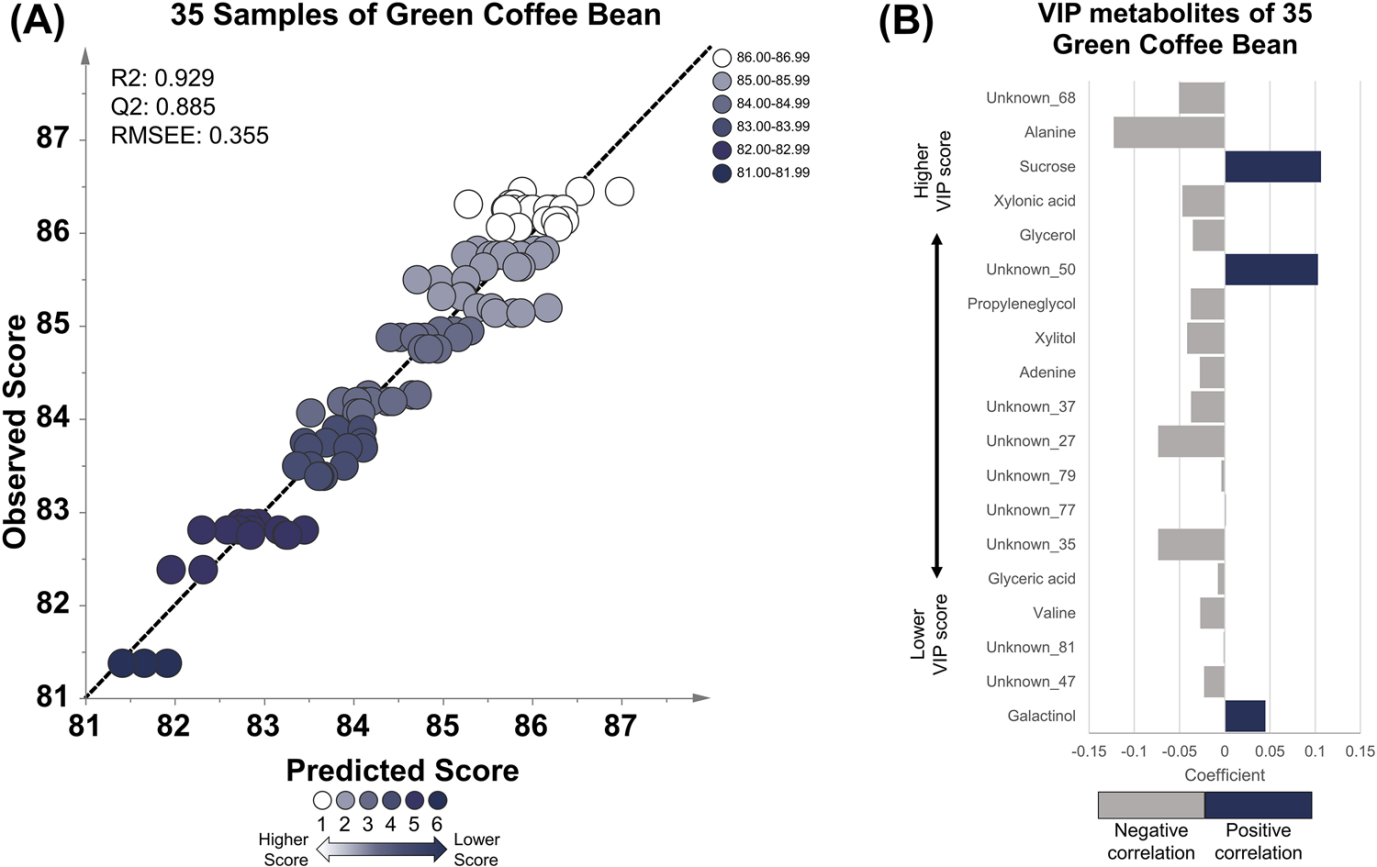
OPLS regression model using green coffee beans of 35 coffee with a score range of 81.38–86.44. **A** The OPLS model shows good linearity and robustness based on the R2, Q2, and RMSEE values. **B** The important metabolites with a VIP value of more than 1.5. Nineteen important metabolites showed positive (blue) and negative correlations (gray). Sucrose, unknown_50, unknown_77, and galactinol correlate positively with cup scores

Galactinol was detected in coffee at different stages, with a consistent correlation with the coffee cup score based on previous OPLS results. Hence, galactinol’s robustness and applicability as a coffee quality marker were further investigated. Moreover, determining coffee quality based on the metabolome profile of green coffee beans could be an efficient screening method. Therefore, OPLS regression analysis was conducted using the metabolite profiles of 35 different kinds of green beans as the validation sample set (Table S1). The final score model for green beans showed high correlation and prediction performance (R2 = 0.96, Q2 = 0.865) (Fig. 5A). The model also shown as significant by CV-ANOVA analysis (Table S5). The result showed that galactinol consistently had a VIP value greater than 1.5 along with other 18 metabolites. Moreover, galactinol was positively correlated with the final cup test score, which is consistent with the previous model (Fig. 5B). The result indicates that galactinol could be used as a quality marker for rapid screening of green coffee beans and complementing cupping tests by skilled panelists.

The study found that galactinol accumulated in green coffee beans, roasted coffee beans, and brewed coffee and was correlated with coffee’s final cup score based on OPLS regression analysis. Hence, galactinol could be suitable as an Arabica specialty coffee quality marker. Since galactinol can be detected early, i.e. in green coffee beans, it has the potential to be used as a parameter for early screening of coffee quality before the cupping test. A validation study using a larger sample set of green coffee beans demonstrated the robustness of galactinol for this purpose. Although galactinol showed a good correlation with coffee quality in terms of the final score, no previous literature describes galactinol’s role in coffee taste in general. This study could be used as a foundation for further studies on galactinol in coffee science in the future.

## Conclusion

A GC/MS-based metabolomics approach was used to determine the potential quality marker candidate for specialty Arabica coffee in three different coffee stages: green coffee beans, roasted coffee beans, and brewed coffee. In this study, the OPLS regression analysis suggested galactinol as a quality marker candidate for Arabica coffee at different coffee production stages (green bean, roasted bean, and brewed coffee). The differences in galactinol concentrations between higher and lower cup scores within the specialty coffee Arabica grade could be detected at an early stage, i.e. in green coffee beans. Hence, there is a possibility that galactinol could be used as a quality maker to provide an early screening method for skilled panelists that could complement the cupping test.

### Supplementary Information

Below is the link to the electronic supplementary material.

Supplementary file1 (DOCX 824 kb)

## Data Availability

The metabolomics and the metadata reported in this paper was submitted to https://www.ebi.ac.uk/metabolights/ with study identifier MTBLS7001.
