# Supplementary material for: Identification of potential quality markers in Indonesia’s Arabica specialty coffee using GC/MS-based metabolomics approach
Source: Metabolomics. 2023 Oct 25;19(11):90. doi: 10.1007/s11306-023-02051-5 (PMC10600306; doi:10.1007/s11306-023-02051-5)
Supplement: Supplementary file 1 — Supplementary file1 (DOCX 824 kb) [file 11306_2023_2051_MOESM1_ESM.docx]

**Identification of potential quality markers in Indonesia’s Arabica specialty coffee using GC/MS-based metabolomics approach**

Fitri Amalia^1^, Tomoya Irifune^1^, Tetsuji Takegami^1^, Yusianto^2^, Ucu Sumirat^2^, Sastia Prama Putri^1,3*^, Eiichiro Fukusaki^1,3,4^

^1^Department of Biotechnology, Graduate School of Engineering, Osaka University, Japan

^2^Indonesian Coffee and Cocoa Research Institute, Indonesia

^3^Industrial Biotechnology Division, Institute for Open and Transdisciplinary Research Initiatives, Osaka University, Japan

^4^Osaka University Shimadzu Omics Innovation Research Laboratories, Japan

*Send correspondence to:

Sastia Prama Putri

E-mail: sastia_putri@bio.eng.osaka-u.ac.jp

Phone/fax: +81-6-6879-7416

**Short title**: Metabolite profiling of specialty coffee Arabica

Table S1 List of Specialty Coffee Arabica Samples

| No | Code | Fragrance /Aroma | Flavor | Aftertaste | Acidity | Body | Uniformity | Balance | Clean cup | Sweetness | Overall | Final score^a^ |
| --- | --- | --- | --- | --- | --- | --- | --- | --- | --- | --- | --- | --- |
| Sample set 1 | | | | | | | | | | | | |
| 1 | A/008 | 8.50 | 8.00 | 7.75 | 8.00 | 8.50 | 10.00 | 8.00 | 10.00 | 10.00 | 8.00 | 86.75 |
| 2 | A/034 | 7.75 | 7.75 | 7.75 | 7.75 | 8.00 | 10.00 | 7.75 | 10.00 | 10.00 | 7.75 | 84.50 |
| 3 | A/009 | 8.00 | 8.00 | 7.75 | 7.50 | 8.00 | 10.00 | 7.50 | 10.00 | 10.00 | 7.50 | 84.25 |
| 4 | A/001 | 7.75 | 7.75 | 7.50 | 7.50 | 7.50 | 10.00 | 7.50 | 10.00 | 10.00 | 7.50 | 83.00 |
| 5 | A/018 | 8.50 | 8.25 | 8.00 | 8.50 | 8.00 | 10.00 | 3.00 | 10.00 | 10.00 | 8.50 | 82.75 |
| 6 | A/026 | 7.75 | 7.50 | 7.50 | 7.50 | 7.50 | 10.00 | 7.50 | 10.00 | 10.00 | 7.50 | 82.75 |
| 7 | A/067 | 7.75 | 7.25 | 7.25 | 7.50 | 8.00 | 10.00 | 7.50 | 10.00 | 10.00 | 7.50 | 82.75 |
| 8 | A/025 | 7.50 | 7.00 | 7.00 | 7.00 | 8.00 | 10.00 | 7.50 | 10.00 | 10.00 | 7.50 | 81.50 |
| 9 | A/063 | 8.00 | 7.25 | 7.00 | 7.00 | 7.25 | 10.00 | 7.00 | 10.00 | 10.00 | 7.00 | 80.50 |
| 10 | A/039 | 7.5 | 7.00 | 7.00 | 7.00 | 7.75 | 10.00 | 7.00 | 10.00 | 10.00 | 7.00 | 80.25 |
|  |  |  |  |  |  |  |  |  |  |  |  |  |
| Sample set 2 | | | | | | | | | | | | |
| 1 | A/097 | 8.00 | 8.06 | 8.00 | 8.13 | 8.06 | 10.00 | 8.06 | 10.00 | 10.00 | 8.13 | 86.44 |
| 2 | A/037 | 7.88 | 8.19 | 8.00 | 8.06 | 8.00 | 10.00 | 8.00 | 10.00 | 10.00 | 8.19 | 86.31 |
| 3 | A/100 | 7.88 | 8.13 | 8.06 | 8.06 | 8.00 | 10.00 | 8.06 | 10.00 | 10.00 | 8.06 | 86.25 |
| 4 | A/013 | 8.00 | 8.13 | 8.00 | 8.19 | 7.94 | 10.00 | 7.88 | 10.00 | 10.00 | 8.13 | 86.25 |
| 5 | A/023 | 7.88 | 7.88 | 7.81 | 7.94 | 8.44 | 10.00 | 8.44 | 10.00 | 10.00 | 7.88 | 86.25 |
| 6 | A/060 | 8.00 | 7.94 | 8.00 | 7.88 | 7.94 | 10.00 | 8.44 | 10.00 | 10.00 | 7.94 | 86.13 |
| 7 | A/038 | 7.94 | 8.00 | 7.94 | 8.19 | 7.94 | 10.00 | 8.00 | 10.00 | 10.00 | 8.06 | 86.06 |
| 8 | A/062 | 8.00 | 7.94 | 8.00 | 8.00 | 7.94 | 10.00 | 7.94 | 10.00 | 10.00 | 8.00 | 85.81 |
| 9 | A/113 | 8.00 | 8.00 | 7.94 | 8.06 | 7.81 | 10.00 | 7.94 | 10.00 | 10.00 | 8.00 | 85.75 |
| 10 | A/110 | 7.81 | 7.88 | 7.94 | 8.13 | 8.06 | 10.00 | 7.94 | 10.00 | 10.00 | 8.00 | 85.75 |
| 11 | A/102 | 8.00 | 7.94 | 8.00 | 8.00 | 7.81 | 10.00 | 7.88 | 10.00 | 10.00 | 8.00 | 85.63 |
| 12 | A/011 | 8.06 | 7.94 | 7.94 | 7.94 | 7.94 | 10.00 | 7.81 | 10.00 | 10.00 | 7.88 | 85.50 |
| 13 | A/065 | 7.94 | 8.06 | 7.88 | 7.94 | 7.81 | 10.00 | 7.81 | 10.00 | 10.00 | 7.88 | 85.31 |
| 14 | A/078 | 7.81 | 8.00 | 7.88 | 7.94 | 7.88 | 10.00 | 7.88 | 10.00 | 10.00 | 7.81 | 85.19 |
| 15 | A/107 | 7.94 | 7.81 | 7.75 | 8.00 | 7.81 | 10.00 | 7.81 | 10.00 | 10.00 | 8.00 | 85.13 |
| 16 | A/061 | 7.88 | 7.94 | 7.75 | 7.94 | 7.75 | 10.00 | 7.81 | 10.00 | 10.00 | 7.88 | 84.94 |
| 17 | A/059 | 8.00 | 7.94 | 7.81 | 7.88 | 7.81 | 10.00 | 7.69 | 10.00 | 10.00 | 7.75 | 84.88 |
| 18 | A/043 | 7.94 | 7.81 | 7.88 | 7.81 | 7.75 | 10.00 | 7.81 | 10.00 | 10.00 | 7.88 | 84.88 |
| 19 | A/112 | 7.88 | 7.81 | 7.75 | 7.94 | 7.88 | 10.00 | 7.75 | 10.00 | 10.00 | 7.75 | 84.75 |
| 20 | A/106 | 7.94 | 7.69 | 7.69 | 7.75 | 7.81 | 10.00 | 7.69 | 10.00 | 10.00 | 7.69 | 84.25 |
| 21 | A/088 | 8.00 | 7.69 | 7.75 | 7.75 | 7.56 | 10.00 | 7.69 | 10.00 | 10.00 | 7.75 | 84.19 |
| 22 | A/091 | 7.81 | 7.75 | 7.75 | 7.75 | 7.81 | 10.00 | 7.63 | 10.00 | 10.00 | 7.69 | 84.19 |
| 23 | A/064 | 7.75 | 7.81 | 7.69 | 7.81 | 7.63 | 10.00 | 7.75 | 10.00 | 10.00 | 7.75 | 84.19 |
| 24 | A/074 | 8.00 | 7.75 | 7.56 | 7.88 | 7.63 | 10.00 | 7.56 | 10.00 | 10.00 | 7.69 | 84.06 |
| 25 | A/056 | 7.75 | 7.81 | 7.63 | 7.75 | 7.69 | 10.00 | 7.63 | 10.00 | 10.00 | 7.63 | 83.88 |
| 26 | A/115 | 7.75 | 7.75 | 7.56 | 7.69 | 7.63 | 10.00 | 7.69 | 10.00 | 10.00 | 7.69 | 83.75 |
| 27 | A/109 | 7.63 | 7.63 | 7.63 | 7.63 | 7.81 | 10.00 | 7.69 | 10.00 | 10.00 | 7.69 | 83.69 |
| 28 | A/044 | 7.63 | 7.56 | 7.75 | 7.56 | 7.75 | 10.00 | 7.63 | 10.00 | 10.00 | 7.63 | 83.50 |
| 29 | A/058 | 7.88 | 7.63 | 7.69 | 7.56 | 7.50 | 10.00 | 7.56 | 10.00 | 10.00 | 7.56 | 83.38 |
| 30 | A/095 | 7.75 | 7.69 | 7.38 | 7.63 | 7.56 | 10.00 | 7.44 | 10.00 | 10.00 | 7.44 | 82.88 |
| 31 | A/024 | 7.56 | 7.50 | 7.56 | 7.50 | 7.69 | 10.00 | 7.50 | 10.00 | 10.00 | 7.50 | 82.81 |
| 32 | A/105 | 7.75 | 7.50 | 7.56 | 7.38 | 7.69 | 10.00 | 7.50 | 10.00 | 10.00 | 7.44 | 82.81 |
| 33 | A/093 | 7.50 | 7.56 | 7.63 | 7.56 | 7.50 | 10.00 | 7.50 | 10.00 | 10.00 | 7.50 | 82.75 |
| 34 | A/017 | 7.56 | 7.50 | 7.50 | 7.25 | 7.69 | 10.00 | 7.44 | 10.00 | 10.00 | 7.44 | 82.38 |
| 35 | A/103 | 7.50 | 7.25 | 7.31 | 7.25 | 7.63 | 10.00 | 7.31 | 10.00 | 10.00 | 7.13 | 81.38 |

^a^ Final scores were calculated by summing scores of all attributes. (The attribute of defects was not recorded in this study.)

Table S2 List of detected metabolites in green beans

| No | Metabolite name | Class | RT^a^ (min) | RI^b^ | Quant mass (*m/z*) | MSI Level^c^ |
| --- | --- | --- | --- | --- | --- | --- |
| 1 | Alanine | Amino Acid | 5.46 | 1108.46 | 116 | 2 |
| 2 | Asparagine | Amino Acid | 10.74 | 1682.93 | 116 | 2 |
| 3 | Aspartic acid | Amino Acid | 9.54 | 1531.00 | 232 | 2 |
| 4 | b-Glutamic acid | Amino Acid | 10.28 | 1622.94 | 232 | 2 |
| 5 | Glutamic acid | Amino Acid | 10.33 | 1629.43 | 246 | 2 |
| 6 | Glutamine | Amino Acid | 11.50 | 1785.39 | 156 | 2 |
| 7 | Glycine | Amino Acid | 7.63 | 1316.70 | 174 | 2 |
| 8 | Isoleucine | Amino Acid | 7.49 | 1301.96 | 158 | 2 |
| 9 | Leucine | Amino Acid | 7.27 | 1280.04 | 158 | 2 |
| 10 | Lysine | Amino Acid | 12.56 | 1939.63 | 156 | 2 |
| 11 | Phenylalanine | Amino Acid | 10.42 | 1641.18 | 218 | 2 |
| 12 | Proline | Amino Acid | 7.52 | 1305.89 | 142 | 2 |
| 13 | Pyroglutamic acid | Amino Acid | 9.55 | 1532.71 | 156 | 2 |
| 14 | Serine | Amino Acid | 7.10 | 1263.26 | 116 | 2 |
| 15 | Threonine | Amino Acid | 8.39 | 1398.21 | 219 | 2 |
| 16 | Tryptophan | Amino Acid | 14.48 | 2247.64 | 202 | 2 |
| 17 | Tyrosine | Amino Acid | 12.68 | 1957.34 | 218 | 2 |
| 18 | Valine | Amino Acid | 6.70 | 1224.12 | 144 | 2 |
| 19 | Chlorogenic acid | Organic Acid | 19.05 | 3173.71 | 345 | 2 |
| 20 | Ferulic acid | Organic Acid | 13.61 | 2102.45 | 338 | 2 |
| 21 | Fumaric acid | Organic Acid | 7.93 | 1348.54 | 245 | 2 |
| 22 | Galacturonic acid | Organic Acid | 13.03 | 2010.30 | 333 | 2 |
| 23 | Glucarate | Organic Acid | 13.34 | 2059.50 | 333 | 2 |
| 24 | Gluconic acid | Organic Acid | 13.24 | 2043.65 | 333 | 2 |
| 25 | Glyceric acid | Organic Acid | 7.85 | 1340.76 | 147 | 2 |
| 26 | Glycolic acid | Organic Acid | 5.14 | 1079.44 | 147 | 2 |
| 27 | Citric acid | Organic Acid | 11.89 | 1840.38 | 273 | 2 |
| 28 | Lactic acid | Organic Acid | 4.98 | 1065.83 | 117 | 2 |
| 29 | Malic acid | Organic Acid | 9.26 | 1498.03 | 147 | 2 |
| 30 | Methyl succinic acid | Organic Acid | 7.84 | 1339.28 | 147 | 2 |
| 31 | Nicotinic acid | Organic Acid | 7.43 | 1296.35 | 180 | 2 |
| 32 | Quinic acid | Organic Acid | 12.24 | 1891.72 | 345 | 2 |
| 33 | Shikimic acid | Organic Acid | 11.76 | 1821.61 | 204 | 2 |
| 34 | Succinic acid | Organic Acid | 7.62 | 1316.32 | 147 | 2 |
| 35 | Xylonic acid | Organic Acid | 11.57 | 1794.54 | 217 | 2 |
| 36 | Fructose | Sugar | 12.40 | 1915.13 | 103 | 2 |
| 37 | Galactose | Sugar | 12.48 | 1927.23 | 319 | 2 |
| 38 | Gentiobiose | Sugar | 17.91 | 2917.30 | 204 | 2 |
| 39 | Glucose | Sugar | 12.52 | 1933.14 | 319 | 2 |
| 40 | Mannose | Sugar | 12.44 | 1920.38 | 319 | 2 |
| 41 | Melezitose | Sugar | 21.36 | 3587.18 | 361 | 2 |
| 42 | Panose | Sugar | 22.88 | 3774.93 | 204 | 2 |
| 43 | Psicose+Tagatose | Sugar | 12.34 | 1905.03 | 103 | 2 |
| 44 | Raffinose | Sugar | 20.80 | 3503.65 | 361 | 2 |
| 45 | Sorbose | Sugar | 12.34 | 1905.04 | 103 | 2 |
| 46 | Sucrose | Sugar | 16.94 | 2710.17 | 361 | 2 |
| 47 | Trehalose | Sugar | 17.46 | 2818.68 | 361 | 2 |
| 48 | Galactose+Glucose | Sugar | 12.65 | 1953.06 | 319 | 2 |
| 49 | Galactinol | Sugar Alcohol | 18.62 | 3077.84 | 204 | 1 |
| 50 | Galactitol | Sugar Alcohol | 12.86 | 1983.59 | 281 | 2 |
| 51 | Inositol | Sugar Alcohol | 13.80 | 2132.87 | 217 | 2 |
| 52 | Mannitol | Sugar Alcohol | 12.76 | 1968.83 | 319 | 2 |
| 53 | Meso erythritol | Sugar Alcohol | 9.49 | 1525.01 | 147 | 2 |
| 54 | Sorbitol | Sugar Alcohol | 12.81 | 1976.58 | 319 | 2 |
| 55 | Threitol | Sugar Alcohol | 9.41 | 1516.34 | 147 | 2 |
| 56 | Xylitol | Sugar Alcohol | 11.13 | 1735.04 | 217 | 2 |
| 57 | 2-Dehydro gluconate | Sugar Derivatives | 12.47 | 1924.57 | 349 | 2 |
| 58 | 2-Aminoethanol | Other | 7.23 | 1276.25 | 174 | 2 |
| 59 | 4-Aminobutyric acid | Other | 9.62 | 1541.52 | 174 | 2 |
| 60 | Adenosine | Other | 16.73 | 2667.59 | 230 | 2 |
| 61 | Caffeine | Other | 12.05 | 1863.08 | 194 | 2 |
| 62 | Glucono-1,5-lactone | Other | 12.37 | 1910.85 | 129 | 2 |
| 63 | Glycerol | Other | 7.30 | 1283.10 | 147 | 2 |
| 64 | N-Methylnicotinate | Other | 9.39 | 1513.55 | 210 | 2 |
| 65 | Paeoniflorin | Other | 20.87 | 3513.86 | 361 | 2 |
| 66 | Phosphate | Other | 7.29 | 1282.14 | 299 | 2 |
| 67 | Putrescine | Other | 11.29 | 1757.53 | 174 | 2 |
| 68 | Serotonin | Other | 15.82 | 2489.52 | 174 | 2 |
| 69 | Unknown_1 | Unknown | 4.68 | 1039.10 | 117 | 4 |
| 70 | Unknown_2 | Organic Acid | 4.78 | 1048.10 | 117 | 3 |
| 71 | Unknown_3 | Unknown | 6.29 | 1184.33 | 281 | 4 |
| 72 | Unknown_4 | Unknown | 6.97 | 1251.14 | 147 | 4 |
| 73 | Unknown_5 | Unknown | 7.11 | 1264.60 | 98 | 4 |
| 74 | Unknown_6 | Organic Acid | 8.43 | 1402.61 | 147 | 3 |
| 75 | Unknown_7 | Organic Acid | 8.66 | 1428.30 | 160 | 3 |
| 76 | Unknown_8 | Unknown | 9.10 | 1479.61 | 210 | 4 |
| 77 | Unknown_9 | Unknown | 9.57 | 1535.39 | 174 | 4 |
| 78 | Unknown_10 | Unknown | 9.63 | 1543.07 | 304 | 4 |
| 79 | Unknown_11 | Unknown | 9.74 | 1555.93 | 120 | 4 |
| 80 | Unknown_12 | Organic Acid | 9.97 | 1583.51 | 129 | 3 |
| 81 | Unknown_13 | Unknown | 10.04 | 1592.21 | 210 | 4 |
| 82 | Unknown_14 | Unknown | 10.12 | 1602.09 | 159 | 4 |
| 83 | Unknown_15 | Unknown | 10.63 | 1668.62 | 355 | 4 |
| 84 | Unknown_16 | Unknown | 11.45 | 1778.43 | 217 | 4 |
| 85 | Unknown_17 | Unknown | 11.67 | 1809.02 | 292 | 4 |
| 86 | Unknown_18 | Unknown | 11.95 | 1848.95 | 204 | 4 |
| 87 | Unknown_19 | Unknown | 12.72 | 1963.69 | 204 | 4 |
| 88 | Unknown_20 | Organic Acid | 12.99 | 2003.92 | 217 | 3 |
| 89 | Unknown_21 | Organic Acid | 13.02 | 2009.13 | 319 | 3 |
| 90 | Unknown_22 | Organic Acid | 13.21 | 2039.22 | 217 | 3 |
| 91 | Unknown_23 | Organic Acid | 13.51 | 2086.76 | 333 | 3 |
| 92 | Unknown_24 | Organic Acid | 13.60 | 2100.89 | 218 | 3 |
| 93 | Unknown_25 | Sugar | 13.81 | 2135.70 | 355 | 3 |
| 94 | Unknown_26 | Unknown | 13.96 | 2160.34 | 290 | 4 |
| 95 | Unknown_27 | Unknown | 14.28 | 2212.92 | 204 | 4 |
| 96 | Unknown_28 | Unknown | 15.39 | 2409.60 | 217 | 4 |
| 97 | Unknown_29 | Unknown | 15.57 | 2443.17 | 204 | 4 |
| 98 | Unknown_30 | Unknown | 16.09 | 2541.39 | 204 | 4 |
| 99 | Unknown_31 | Unknown | 16.76 | 2674.24 | 259 | 4 |
| 100 | Unknown_32 | Unknown | 17.02 | 2725.71 | 191 | 4 |
| 101 | Unknown_33 | Unknown | 17.35 | 2795.82 | 191 | 4 |
| 102 | Unknown_34 | Unknown | 17.83 | 2899.14 | 191 | 4 |
| 103 | Unknown_35 | Unknown | 18.25 | 2991.30 | 355 | 4 |
| 104 | Unknown_36 | Unknown | 18.30 | 3002.06 | 355 | 4 |
| 105 | Unknown_37 | Unknown | 18.36 | 3016.01 | 355 | 4 |
| 106 | Unknown_38 | Unknown | 18.39 | 3024.43 | 345 | 4 |
| 107 | Unknown_39 | Unknown | 18.45 | 3037.82 | 355 | 4 |
| 108 | Unknown_40 | Unknown | 18.74 | 3104.34 | 219 | 4 |
| 109 | Unknown_41 | Unknown | 18.78 | 3113.19 | 204 | 4 |
| 110 | Unknown_42 | Unknown | 18.82 | 3121.59 | 204 | 4 |
| 111 | Unknown_43 | Unknown | 18.92 | 3145.18 | 345 | 4 |
| 112 | Unknown_44 | Unknown | 19.01 | 3163.63 | 207 | 4 |
| 113 | Unknown_45 | Unknown | 19.16 | 3198.69 | 204 | 4 |
| 114 | Unknown_46 | Unknown | 19.18 | 3201.05 | 249 | 4 |
| 115 | Unknown_47 | Unknown | 19.27 | 3219.91 | 307 | 4 |
| 116 | Unknown_48 | Unknown | 19.30 | 3227.10 | 249 | 4 |
| 117 | Unknown_49 | Unknown | 19.33 | 3233.57 | 207 | 4 |
| 118 | Unknown_50 | Unknown | 19.38 | 3242.47 | 307 | 4 |
| 119 | Unknown_51 | Unknown | 20.67 | 3482.95 | 255 | 4 |
| 120 | Unknown_52 | Unknown | 20.73 | 3491.92 | 255 | 4 |
| 121 | Unknown_53 | Unknown | 21.09 | 3546.40 | 361 | 4 |
| 122 | Unknown_54 | Unknown | 21.56 | 3615.61 | 362 | 4 |
| 123 | Unknown_55 | Sugar | 21.57 | 3615.75 | 361 | 3 |
| 124 | Unknown_56 | Unknown | 21.64 | 3625.13 | 361 | 4 |

There are 124 metabolites detected in all green bean samples

a Retention time in minute(s)

b Retention indices (RI) are calculated using a standard alkane mixture (C10–C40).

c The MSI confidence level of annotation and identification. Mass spectra of metabolite peaks that are compared with the library and the authentic standard considered as Metabolic Standards Initiative (MSI) level 1. Mass spectra of metabolite peaks only with the in-house library are considered MSI level 2 for putatively annotated compound and MSI level 3 for putatively characterized compound classes. The metabolite peak categorized as MSI level 4 if there are no similarities with any chemical class compound in library (Sumner et al. 2007).

Table S3 List of detected metabolites in roasted beans

| No | Metabolite name | Class | RT^a^ (min) | RI^b^ | Quant mass (*m/z*) | MSI Level^c^ |
| --- | --- | --- | --- | --- | --- | --- |
| 1 | 5-Oxoproline | Amino Acid | 9.48 | 1531.80 | 156 | 2 |
| 2 | Aspartic acid | Amino Acid | 9.48 | 1531.00 | 232 | 2 |
| 3 | Lauric acid | Fatty Acid | 10.45 | 1652.17 | 117 | 2 |
| 4 | Myristic acid | Fatty Acid | 11.89 | 1848.96 | 117 | 2 |
| 5 | Plamitic acid | Fatty Acid | 13.19 | 2045.77 | 117 | 2 |
| 6 | Stearic acid | Fatty Acid | 14.39 | 2243.20 | 117 | 2 |
| 7 | Chlorogenic acid | Organic Acid | 18.96 | 3169.59 | 345 | 2 |
| 8 | Fumaric acid | Organic Acid | 7.87 | 1348.21 | 245 | 2 |
| 9 | Galacturonic acid | Organic Acid | 12.96 | 2009.35 | 333 | 2 |
| 10 | Glucarate | Organic Acid | 12.93 | 2003.44 | 217 | 2 |
| 11 | Gluconic acid | Organic Acid | 13.17 | 2042.56 | 333 | 2 |
| 12 | Glyceric acid | Organic Acid | 7.79 | 1340.29 | 147 | 2 |
| 13 | Glycolic acid | Organic Acid | 5.08 | 1078.70 | 147 | 2 |
| 14 | Citric acid | Organic Acid | 11.82 | 1839.53 | 273 | 2 |
| 15 | Lactic acid | Organic Acid | 4.92 | 1065.05 | 147 | 2 |
| 16 | Maleic acid | Organic Acid | 7.49 | 1307.89 | 147 | 2 |
| 17 | Malic acid | Organic Acid | 9.20 | 1497.45 | 147 | 2 |
| 18 | Methyl succinic acid | Organic Acid | 7.69 | 1329.34 | 147 | 2 |
| 19 | Nicotinic acid | Organic Acid | 7.37 | 1295.46 | 180 | 2 |
| 20 | Quinic acid | Organic Acid | 12.18 | 1891.26 | 345 | 2 |
| 21 | Shikimic acid | Organic Acid | 11.69 | 1821.02 | 204 | 2 |
| 22 | Succinic acid | Organic Acid | 7.56 | 1315.70 | 147 | 2 |
| 23 | Allose | Sugar | 12.33 | 1912.52 | 319 | 2 |
| 24 | Arabionose | Sugar | 10.71 | 1686.27 | 103 | 2 |
| 25 | b-Lactose | Sugar | 17.22 | 2782.30 | 361 | 2 |
| 26 | Fructose | Sugar | 12.34 | 1914.50 | 103 | 2 |
| 27 | Galactose | Sugar | 12.45 | 1932.05 | 147 | 2 |
| 28 | Galactose+Glucose | Sugar | 12.59 | 1952.82 | 319 | 2 |
| 29 | Glucose | Sugar | 12.46 | 1932.45 | 319 | 2 |
| 30 | Lyxose | Sugar | 10.71 | 1685.70 | 103 | 2 |
| 31 | Maltose | Sugar | 17.41 | 2822.33 | 169 | 2 |
| 32 | Mannose | Sugar | 12.37 | 1919.77 | 319 | 2 |
| 33 | Melezitose | Sugar | 21.28 | 3594.63 | 361 | 2 |
| 34 | Melibiose | Sugar | 17.98 | 2946.59 | 204 | 2 |
| 35 | Panose | Sugar | 22.70 | 3773.28 | 204 | 2 |
| 36 | Psicose | Sugar | 12.17 | 1890.16 | 103 | 2 |
| 37 | Psicose+Tagatose | Sugar | 12.27 | 1904.42 | 103 | 2 |
| 38 | Rhamnose | Sugar | 11.14 | 1744.95 | 117 | 2 |
| 39 | Ribose | Sugar | 10.82 | 1700.70 | 103 | 2 |
| 40 | Sucrose | Sugar | 16.85 | 2704.40 | 361 | 2 |
| 41 | Tagatose | Sugar | 12.12 | 1882.31 | 103 | 2 |
| 42 | Trehalose | Sugar | 17.41 | 2821.66 | 361 | 2 |
| 43 | Turanose | Sugar | 17.45 | 2830.16 | 217 | 2 |
| 44 | Xylulose+Ribulose | Sugar | 10.82 | 1700.46 | 147 | 2 |
| 45 | Allose+Mannose | Sugar | 12.50 | 1939.14 | 147 | 2 |
| 46 | Arabitol | Sugar Alcohol | 11.16 | 1746.67 | 217 | 2 |
| 47 | Galactinol | Sugar Alcohol | 18.55 | 3075.72 | 204 | 2 |
| 48 | Inositol | Sugar Alcohol | 13.73 | 2131.82 | 217 | 2 |
| 49 | Maltitol | Sugar Alcohol | 17.93 | 2935.27 | 204 | 2 |
| 50 | Mannitol | Sugar Alcohol | 12.69 | 1968.11 | 319 | 2 |
| 51 | Meso erythritol | Sugar Alcohol | 9.43 | 1524.55 | 217 | 2 |
| 52 | Sorbitol | Sugar Alcohol | 12.74 | 1975.77 | 319 | 2 |
| 53 | Xylitol | Sugar Alcohol | 11.07 | 1734.28 | 217 | 2 |
| 54 | Threonic acid | Sugar Derivatives | 9.86 | 1577.02 | 292 | 2 |
| 55 | 1,6-Anhydroglucose | Other | 10.99 | 1724.36 | 217 | 2 |
| 56 | 2-Hydroxybutyrate | Other | 5.68 | 1133.28 | 131 | 2 |
| 57 | 2-Hydroxypyridine | Other | 4.63 | 1039.15 | 152 | 2 |
| 58 | 3,4-Dihydroxybenzoate | Other | 11.77 | 1832.17 | 193 | 2 |
| 59 | 3-Hydroxybenzoate | Other | 9.81 | 1571.50 | 267 | 2 |
| 60 | 4-Aminobutyric acid | Other | 9.56 | 1540.84 | 174 | 2 |
| 61 | Adenine | Other | 12.11 | 1881.53 | 264 | 2 |
| 62 | Caffeine | Other | 11.98 | 1862.30 | 194 | 2 |
| 63 | Catechol | Other | 7.66 | 1326.44 | 254 | 2 |
| 64 | Cytosine | Other | 9.57 | 1542.50 | 254 | 2 |
| 65 | Dihydroxyacetone | Other | 7.00 | 1259.29 | 103 | 2 |
| 66 | Glucono-1,5-lactone | Other | 12.35 | 1916.20 | 255 | 2 |
| 67 | Glycerol | Other | 7.24 | 1282.68 | 147 | 2 |
| 68 | N-Methylnicotinate | Other | 9.33 | 1512.61 | 210 | 2 |
| 69 | Oxalacetic acid+Pyruvate | Other | 4.77 | 1051.48 | 174 | 2 |
| 70 | Phosphate | Other | 7.23 | 1281.57 | 299 | 2 |
| 71 | Propyleneglycol | Other | 4.25 | 1004.65 | 117 | 2 |
| 72 | Pyrogallol | Other | 9.71 | 1558.73 | 239 | 2 |
| 73 | Unknown_1 | Unknown | 4.07 | 981.49 | 147 | 4 |
| 74 | Unknown_2 | Unknown | 4.19 | 999.21 | 155 | 4 |
| 75 | Unknown_3 | Unknown | 4.63 | 1038.49 | 117 | 4 |
| 76 | Unknown_4 | Unknown | 4.73 | 1047.48 | 117 | 4 |
| 77 | Unknown_5 | Unknown | 5.38 | 1105.40 | 89 | 4 |
| 78 | Unknown_6 | Unknown | 5.63 | 1129.05 | 89 | 4 |
| 79 | Unknown_7 | Unknown | 5.71 | 1136.35 | 125 | 4 |
| 80 | Unknown_8 | Unknown | 5.75 | 1139.59 | 152 | 4 |
| 81 | Unknown_9 | Unknown | 5.82 | 1146.12 | 147 | 4 |
| 82 | Unknown_10 | Unknown | 5.83 | 1146.93 | 142 | 4 |
| 83 | Unknown_11 | Unknown | 5.84 | 1147.97 | 177 | 4 |
| 84 | Unknown_12 | Unknown | 6.06 | 1168.35 | 89 | 4 |
| 85 | Unknown_13 | Unknown | 6.07 | 1169.19 | 131 | 4 |
| 86 | Unknown_14 | Unknown | 6.17 | 1178.85 | 132 | 4 |
| 87 | Unknown_15 | Unknown | 6.30 | 1190.57 | 166 | 4 |
| 88 | Unknown_16 | Unknown | 6.35 | 1195.00 | 132 | 4 |
| 89 | Unknown_17 | Unknown | 6.42 | 1202.08 | 166 | 4 |
| 90 | Unknown_18 | Unknown | 6.60 | 1219.20 | 117 | 4 |
| 91 | Unknown_19 | Unknown | 6.78 | 1237.78 | 116 | 4 |
| 92 | Unknown_20 | Unknown | 6.81 | 1240.27 | 156 | 4 |
| 93 | Unknown_21 | Unknown | 6.82 | 1241.52 | 174 | 4 |
| 94 | Unknown_22 | Unknown | 6.91 | 1250.56 | 147 | 4 |
| 95 | Unknown_23 | Unknown | 6.93 | 1252.14 | 96 | 4 |
| 96 | Unknown_24 | Unknown | 7.05 | 1263.77 | 98 | 4 |
| 97 | Unknown_25 | Unknown | 7.22 | 1280.78 | 300 | 4 |
| 98 | Unknown_26 | Unknown | 7.23 | 1282.06 | 133 | 4 |
| 99 | Unknown_27 | Unknown | 7.33 | 1291.76 | 183 | 4 |
| 100 | Unknown_28 | Unknown | 7.37 | 1296.00 | 129 | 4 |
| 101 | Unknown_29 | Unknown | 7.44 | 1302.47 | 117 | 4 |
| 102 | Unknown_30 | Unknown | 7.44 | 1302.80 | 169 | 4 |
| 103 | Unknown_31 | Unknown | 7.85 | 1346.99 | 147 | 4 |
| 104 | Unknown_32 | Unknown | 7.86 | 1347.62 | 133 | 4 |
| 105 | Unknown_33 | Unknown | 7.94 | 1356.14 | 147 | 4 |
| 106 | Unknown_34 | Unknown | 8.03 | 1366.00 | 147 | 4 |
| 107 | Unknown_35 | Unknown | 8.20 | 1383.81 | 147 | 4 |
| 108 | Unknown_36 | Unknown | 8.22 | 1386.38 | 94 | 4 |
| 109 | Unknown_37 | Unknown | 8.33 | 1397.90 | 89 | 4 |
| 110 | Unknown_38 | Unknown | 8.35 | 1400.04 | 259 | 4 |
| 111 | Unknown_39 | Unknown | 8.37 | 1401.98 | 147 | 4 |
| 112 | Unknown_40 | Unknown | 8.38 | 1402.92 | 89 | 4 |
| 113 | Unknown_41 | Unknown | 8.38 | 1403.55 | 138 | 4 |
| 114 | Unknown_42 | Unknown | 8.39 | 1403.94 | 106 | 4 |
| 115 | Unknown_43 | Unknown | 8.41 | 1407.07 | 239 | 4 |
| 116 | Unknown_44 | Unknown | 8.44 | 1410.08 | 100 | 4 |
| 117 | Unknown_45 | Unknown | 8.47 | 1413.14 | 217 | 4 |
| 118 | Unknown_46 | Unknown | 8.53 | 1420.11 | 169 | 4 |
| 119 | Unknown_47 | Unknown | 8.54 | 1421.45 | 103 | 4 |
| 120 | Unknown_48 | Unknown | 8.67 | 1435.94 | 89 | 4 |
| 121 | Unknown_49 | Unknown | 8.71 | 1440.70 | 147 | 4 |
| 122 | Unknown_50 | Unknown | 8.72 | 1442.17 | 174 | 4 |
| 123 | Unknown_51 | Unknown | 8.97 | 1470.88 | 147 | 4 |
| 124 | Unknown_52 | Unknown | 9.03 | 1478.14 | 218 | 4 |
| 125 | Unknown_53 | Unknown | 9.04 | 1478.75 | 210 | 4 |
| 126 | Unknown_54 | Unknown | 9.07 | 1482.51 | 103 | 4 |
| 127 | Unknown_55 | Unknown | 9.16 | 1492.76 | 103 | 4 |
| 128 | Unknown_56 | Unknown | 9.18 | 1494.76 | 271 | 4 |
| 129 | Unknown_57 | Unknown | 9.23 | 1500.70 | 254 | 4 |
| 130 | Unknown_58 | Unknown | 9.24 | 1501.73 | 157 | 4 |
| 131 | Unknown_59 | Unknown | 9.28 | 1507.32 | 143 | 4 |
| 132 | Unknown_60 | Unknown | 9.28 | 1507.35 | 117 | 4 |
| 133 | Unknown_61 | Unknown | 9.36 | 1516.23 | 217 | 4 |
| 134 | Unknown_62 | Unknown | 9.36 | 1516.53 | 143 | 4 |
| 135 | Unknown_63 | Unknown | 9.37 | 1517.39 | 103 | 4 |
| 136 | Unknown_64 | Unknown | 9.39 | 1519.97 | 174 | 4 |
| 137 | Unknown_65 | Unknown | 9.44 | 1526.33 | 117 | 4 |
| 138 | Unknown_66 | Unknown | 9.51 | 1535.37 | 237 | 4 |
| 139 | Unknown_67 | Unknown | 9.63 | 1549.37 | 101 | 4 |
| 140 | Unknown_68 | Unknown | 9.67 | 1554.29 | 147 | 4 |
| 141 | Unknown_69 | Unknown | 9.73 | 1561.49 | 292 | 4 |
| 142 | Unknown_70 | Unknown | 9.80 | 1570.37 | 223 | 4 |
| 143 | Unknown_71 | Unknown | 9.81 | 1570.88 | 143 | 4 |
| 144 | Unknown_72 | Unknown | 9.88 | 1579.56 | 174 | 4 |
| 145 | Unknown_73 | Unknown | 9.97 | 1591.41 | 210 | 4 |
| 146 | Unknown_74 | Unknown | 9.99 | 1593.55 | 103 | 4 |
| 147 | Unknown_75 | Unknown | 9.99 | 1593.70 | 147 | 4 |
| 148 | Unknown_76 | Unknown | 10.00 | 1595.14 | 174 | 4 |
| 149 | Unknown_77 | Unknown | 10.11 | 1608.44 | 253 | 4 |
| 150 | Unknown_78 | Unknown | 10.13 | 1611.33 | 156 | 4 |
| 151 | Unknown_79 | Unknown | 10.15 | 1614.32 | 342 | 4 |
| 152 | Unknown_80 | Unknown | 10.19 | 1619.17 | 239 | 4 |
| 153 | Unknown_81 | Unknown | 10.30 | 1632.97 | 217 | 4 |
| 154 | Unknown_82 | Unknown | 10.30 | 1633.35 | 147 | 4 |
| 155 | Unknown_83 | Unknown | 10.44 | 1650.86 | 147 | 4 |
| 156 | Unknown_84 | Unknown | 10.55 | 1665.29 | 245 | 4 |
| 157 | Unknown_85 | Unknown | 10.57 | 1668.09 | 355 | 4 |
| 158 | Unknown_86 | Unknown | 10.66 | 1679.35 | 103 | 4 |
| 159 | Unknown_87 | Unknown | 10.66 | 1680.30 | 245 | 4 |
| 160 | Unknown_88 | Unknown | 10.84 | 1703.07 | 102 | 4 |
| 161 | Unknown_89 | Unknown | 10.87 | 1707.47 | 268 | 4 |
| 162 | Unknown_90 | Unknown | 10.88 | 1708.55 | 117 | 4 |
| 163 | Unknown_91 | Unknown | 10.88 | 1709.03 | 143 | 4 |
| 164 | Unknown_92 | Unknown | 10.90 | 1711.15 | 209 | 4 |
| 165 | Unknown_93 | Unknown | 10.97 | 1721.11 | 311 | 4 |
| 166 | Unknown_94 | Unknown | 11.07 | 1734.62 | 103 | 4 |
| 167 | Unknown_95 | Unknown | 11.27 | 1762.34 | 207 | 4 |
| 168 | Unknown_96 | Unknown | 11.33 | 1770.66 | 147 | 4 |
| 169 | Unknown_97 | Unknown | 11.34 | 1772.12 | 231 | 4 |
| 170 | Unknown_98 | Unknown | 11.37 | 1775.91 | 169 | 4 |
| 171 | Unknown_99 | Unknown | 11.38 | 1776.80 | 231 | 4 |
| 172 | Unknown_100 | Unknown | 11.39 | 1777.96 | 217 | 4 |
| 173 | Unknown_101 | Unknown | 11.39 | 1778.21 | 147 | 4 |
| 174 | Unknown_102 | Unknown | 11.47 | 1789.70 | 325 | 4 |
| 175 | Unknown_103 | Unknown | 11.49 | 1792.43 | 129 | 4 |
| 176 | Unknown_104 | Unknown | 11.53 | 1798.29 | 334 | 4 |
| 177 | Unknown_105 | Unknown | 11.54 | 1798.50 | 129 | 4 |
| 178 | Unknown_106 | Unknown | 11.60 | 1807.48 | 246 | 4 |
| 179 | Unknown_107 | Unknown | 11.60 | 1807.83 | 147 | 4 |
| 180 | Unknown_108 | Unknown | 11.63 | 1811.33 | 231 | 4 |
| 181 | Unknown_109 | Unknown | 11.64 | 1812.80 | 204 | 4 |
| 182 | Unknown_110 | Unknown | 11.72 | 1825.49 | 187 | 4 |
| 183 | Unknown_111 | Unknown | 11.75 | 1828.68 | 147 | 4 |
| 184 | Unknown_112 | Unknown | 11.79 | 1835.19 | 204 | 4 |
| 185 | Unknown_113 | Unknown | 11.82 | 1839.47 | 375 | 4 |
| 186 | Unknown_114 | Unknown | 11.84 | 1842.01 | 195 | 4 |
| 187 | Unknown_115 | Unknown | 12.01 | 1866.74 | 345 | 4 |
| 188 | Unknown_116 | Unknown | 12.04 | 1870.50 | 129 | 4 |
| 189 | Unknown_117 | Unknown | 12.08 | 1876.67 | 245 | 4 |
| 190 | Unknown_118 | Unknown | 12.11 | 1880.71 | 245 | 4 |
| 191 | Unknown_119 | Unknown | 12.15 | 1886.47 | 245 | 4 |
| 192 | Unknown_120 | Unknown | 12.18 | 1891.22 | 346 | 4 |
| 193 | Unknown_121 | Unknown | 12.18 | 1891.34 | 115 | 4 |
| 194 | Unknown_122 | Unknown | 12.25 | 1901.26 | 147 | 4 |
| 195 | Unknown_123 | Unknown | 12.28 | 1905.42 | 240 | 4 |
| 196 | Unknown_124 | Unknown | 12.46 | 1932.31 | 320 | 4 |
| 197 | Unknown_125 | Unknown | 12.50 | 1939.57 | 116 | 4 |
| 198 | Unknown_126 | Unknown | 12.53 | 1943.82 | 148 | 4 |
| 199 | Unknown_127 | Unknown | 12.55 | 1946.87 | 345 | 4 |
| 200 | Unknown_128 | Unknown | 12.59 | 1953.08 | 147 | 4 |
| 201 | Unknown_129 | Unknown | 12.60 | 1954.83 | 315 | 4 |
| 202 | Unknown_130 | Unknown | 12.63 | 1958.71 | 345 | 4 |
| 203 | Unknown_131 | Unknown | 12.64 | 1960.19 | 179 | 4 |
| 204 | Unknown_132 | Unknown | 12.66 | 1963.22 | 204 | 4 |
| 205 | Unknown_133 | Unknown | 12.73 | 1973.01 | 345 | 4 |
| 206 | Unknown_134 | Unknown | 12.79 | 1982.79 | 147 | 4 |
| 207 | Unknown_135 | Unknown | 12.84 | 1990.41 | 175 | 4 |
| 208 | Unknown_136 | Unknown | 12.87 | 1994.84 | 204 | 4 |
| 209 | Unknown_137 | Unknown | 12.97 | 2010.88 | 281 | 4 |
| 210 | Unknown_138 | Unknown | 13.02 | 2018.85 | 281 | 4 |
| 211 | Unknown_139 | Unknown | 13.15 | 2038.03 | 147 | 4 |
| 212 | Unknown_140 | Unknown | 13.15 | 2038.33 | 217 | 4 |
| 213 | Unknown_141 | Unknown | 13.19 | 2045.45 | 313 | 4 |
| 214 | Unknown_142 | Unknown | 13.45 | 2085.48 | 333 | 4 |
| 215 | Unknown_143 | Unknown | 13.73 | 2131.66 | 147 | 4 |
| 216 | Unknown_144 | Unknown | 13.73 | 2132.30 | 318 | 4 |
| 217 | Unknown_145 | Unknown | 13.75 | 2134.51 | 355 | 4 |
| 218 | Unknown_146 | Unknown | 14.07 | 2187.75 | 204 | 4 |
| 219 | Unknown_147 | Unknown | 14.22 | 2212.71 | 204 | 4 |
| 220 | Unknown_148 | Unknown | 14.30 | 2226.80 | 204 | 4 |
| 221 | Unknown_149 | Unknown | 14.41 | 2246.48 | 204 | 4 |
| 222 | Unknown_150 | Unknown | 15.02 | 2353.30 | 204 | 4 |
| 223 | Unknown_151 | Unknown | 15.19 | 2384.34 | 217 | 4 |
| 224 | Unknown_152 | Unknown | 15.33 | 2408.83 | 217 | 4 |
| 225 | Unknown_153 | Unknown | 15.45 | 2432.39 | 217 | 4 |
| 226 | Unknown_154 | Unknown | 15.46 | 2434.28 | 204 | 4 |
| 227 | Unknown_155 | Unknown | 15.87 | 2510.79 | 345 | 4 |
| 228 | Unknown_156 | Unknown | 16.02 | 2540.44 | 204 | 4 |
| 229 | Unknown_157 | Unknown | 16.08 | 2550.54 | 217 | 4 |
| 230 | Unknown_158 | Unknown | 16.11 | 2556.41 | 259 | 4 |
| 231 | Unknown_159 | Unknown | 16.11 | 2557.42 | 217 | 4 |
| 232 | Unknown_160 | Unknown | 16.25 | 2585.05 | 259 | 4 |
| 233 | Unknown_161 | Unknown | 16.31 | 2595.71 | 371 | 4 |
| 234 | Unknown_162 | Unknown | 16.32 | 2597.64 | 217 | 4 |
| 235 | Unknown_163 | Unknown | 16.38 | 2610.02 | 217 | 4 |
| 236 | Unknown_164 | Unknown | 16.64 | 2661.86 | 217 | 4 |
| 237 | Unknown_165 | Unknown | 16.70 | 2674.54 | 361 | 4 |
| 238 | Unknown_166 | Unknown | 16.70 | 2674.70 | 217 | 4 |
| 239 | Unknown_167 | Unknown | 16.74 | 2681.63 | 361 | 4 |
| 240 | Unknown_168 | Unknown | 16.85 | 2703.66 | 360 | 4 |
| 241 | Unknown_169 | Unknown | 16.92 | 2718.69 | 103 | 4 |
| 242 | Unknown_170 | Unknown | 16.94 | 2724.18 | 191 | 4 |
| 243 | Unknown_171 | Unknown | 16.95 | 2725.42 | 139 | 4 |
| 244 | Unknown_172 | Unknown | 17.00 | 2735.63 | 361 | 4 |
| 245 | Unknown_173 | Unknown | 17.09 | 2755.46 | 271 | 4 |
| 246 | Unknown_174 | Unknown | 17.17 | 2771.08 | 204 | 4 |
| 247 | Unknown_175 | Unknown | 17.20 | 2776.98 | 217 | 4 |
| 248 | Unknown_176 | Unknown | 17.24 | 2786.80 | 319 | 4 |
| 249 | Unknown_177 | Unknown | 17.29 | 2796.66 | 156 | 4 |
| 250 | Unknown_178 | Unknown | 17.49 | 2839.82 | 204 | 4 |
| 251 | Unknown_179 | Unknown | 17.50 | 2842.27 | 345 | 4 |
| 252 | Unknown_180 | Unknown | 17.51 | 2842.80 | 255 | 4 |
| 253 | Unknown_181 | Unknown | 17.66 | 2875.13 | 345 | 4 |
| 254 | Unknown_182 | Unknown | 17.69 | 2883.41 | 217 | 4 |
| 255 | Unknown_183 | Unknown | 17.70 | 2885.47 | 204 | 4 |
| 256 | Unknown_184 | Unknown | 17.76 | 2897.48 | 191 | 4 |
| 257 | Unknown_185 | Unknown | 17.78 | 2901.31 | 217 | 4 |
| 258 | Unknown_186 | Unknown | 17.80 | 2906.20 | 217 | 4 |
| 259 | Unknown_187 | Unknown | 17.84 | 2915.81 | 204 | 4 |
| 260 | Unknown_188 | Unknown | 18.24 | 3003.66 | 105 | 4 |
| 261 | Unknown_189 | Unknown | 18.70 | 3111.60 | 204 | 4 |
| 262 | Unknown_190 | Unknown | 18.74 | 3120.05 | 204 | 4 |
| 263 | Unknown_191 | Unknown | 19.08 | 3196.34 | 204 | 4 |
| 264 | Unknown_192 | Unknown | 19.18 | 3217.97 | 308 | 4 |
| 265 | Unknown_193 | Unknown | 19.18 | 3218.55 | 307 | 4 |
| 266 | Unknown_194 | Unknown | 19.29 | 3241.10 | 307 | 4 |
| 267 | Unknown_195 | Unknown | 19.63 | 3312.09 | 217 | 4 |
| 268 | Unknown_196 | Unknown | 19.89 | 3361.30 | 361 | 4 |
| 269 | Unknown_197 | Unknown | 19.96 | 3376.67 | 307 | 4 |
| 270 | Unknown_198 | Unknown | 20.10 | 3402.00 | 217 | 4 |
| 271 | Unknown_199 | Unknown | 20.27 | 3432.88 | 204 | 4 |
| 272 | Unknown_200 | Unknown | 20.36 | 3446.98 | 441 | 4 |
| 273 | Unknown_201 | Unknown | 20.74 | 3512.59 | 361 | 4 |
| 274 | Unknown_202 | Unknown | 20.75 | 3513.78 | 217 | 4 |
| 275 | Unknown_203 | Unknown | 21.21 | 3584.44 | 361 | 4 |
| 276 | Unknown_204 | Unknown | 21.48 | 3621.92 | 361 | 4 |
| 277 | Unknown_205 | Unknown | 22.70 | 3773.20 | 191 | 4 |
| 278 | Unknown_206 | Unknown | 22.70 | 3773.34 | 447 | 4 |
| 279 | Unknown_207 | Unknown | 22.70 | 3773.46 | 103 | 4 |

There are 279 metabolites detected in all roasted bean samples

a Retention time in minute(s)

b Retention indices (RI) are calculated using a standard alkane mixture (C10–C40).

c The MSI confidence level of annotation and identification. Mass spectra of metabolite peaks that are compared with the library and the authentic standard considered as Metabolic Standards Initiative (MSI) level 1. Mass spectra of metabolite peaks only with the in-house library are considered MSI level 2 for putatively annotated compound and MSI level 3 for putatively characterized compound classes. The metabolite peak categorized as MSI level 4 if there are no similarities with any chemical class compound in library (Sumner et al. 2007).

Table S4 List of detected metabolites in brewed coffee

| No | Metabolite name | Class | RT^a^ (min) | RI^b^ | Quant mass (*m/z*) | MSI Level^c^ |
| --- | --- | --- | --- | --- | --- | --- |
| 1 | Pyroglutamic acid | Amino acid | 9.41 | 1534.04 | 156 | 2 |
| 2 | Icosanoic acid | Fatty Acid | 15.45 | 2443.18 | 117 | 2 |
| 3 | Myristic acid | Fatty Acid | 11.82 | 1850.42 | 117 | 2 |
| 4 | Oleic acid | Fatty Acid | 14.19 | 2221.16 | 117 | 2 |
| 5 | Plamitic acid | Fatty Acid | 13.13 | 2047.56 | 117 | 2 |
| 6 | Stearic acid | Fatty Acid | 14.34 | 2245.27 | 117 | 2 |
| 7 | 9,12-Octadecadienoic acid | Organic Acid | 14.17 | 2217.40 | 95 | 2 |
| 8 | Altronic acid | Organic Acid | 12.86 | 2005.04 | 217 | 2 |
| 9 | Chlorogenic acid | Organic Acid | 18.91 | 3171.04 | 345 | 2 |
| 10 | Fumaric acid | Organic Acid | 7.78 | 1349.30 | 245 | 2 |
| 11 | Galacturonic acid | Organic Acid | 12.89 | 2009.90 | 333 | 2 |
| 12 | Gluconic acid | Organic Acid | 13.11 | 2043.62 | 333 | 2 |
| 13 | Glyceric acid | Organic Acid | 7.71 | 1341.37 | 147 | 2 |
| 14 | Glycolic acid | Organic Acid | 4.97 | 1078.72 | 147 | 2 |
| 15 | Citric acid | Organic Acid | 11.75 | 1840.98 | 273 | 2 |
| 16 | Lactic acid | Organic Acid | 4.81 | 1064.93 | 147 | 2 |
| 17 | Maleic acid | Organic Acid | 7.41 | 1309.71 | 147 | 2 |
| 18 | Malic acid | Organic Acid | 9.12 | 1498.76 | 147 | 2 |
| 19 | Methyl maleic acid | Organic Acid | 7.86 | 1358.05 | 147 | 2 |
| 20 | Methyl succinic acid | Organic Acid | 7.61 | 1330.71 | 147 | 2 |
| 21 | Nicotinic acid | Organic Acid | 7.29 | 1297.02 | 180 | 2 |
| 22 | Quinic acid | Organic Acid | 12.11 | 1891.88 | 345 | 2 |
| 23 | Shikimic acid | Organic Acid | 11.62 | 1822.75 | 204 | 2 |
| 24 | Succinic acid | Organic Acid | 7.48 | 1317.16 | 147 | 2 |
| 25 | Allose | Sugar | 12.25 | 1913.30 | 319 | 2 |
| 26 | Allose+Mannose | Sugar | 12.43 | 1939.86 | 147 | 2 |
| 27 | Arabinose | Sugar | 10.63 | 1686.90 | 103 | 2 |
| 28 | b-Lactose | Sugar | 17.18 | 2785.00 | 361 | 2 |
| 29 | Fructose | Sugar | 12.27 | 1915.48 | 217 | 2 |
| 30 | Galactinol | Sugar | 18.50 | 3076.69 | 204 | 2 |
| 31 | Galactose | Sugar | 12.34 | 1927.13 | 319 | 2 |
| 32 | Galactose+Glucose | Sugar | 12.52 | 1953.50 | 147 | 2 |
| 33 | Glucose | Sugar | 12.39 | 1933.48 | 147 | 2 |
| 34 | Lyxose | Sugar | 10.58 | 1681.03 | 217 | 2 |
| 35 | Maltose | Sugar | 17.36 | 2824.97 | 169 | 2 |
| 36 | Mannose | Sugar | 12.30 | 1920.72 | 147 | 2 |
| 37 | Panose | Sugar | 22.66 | 3775.44 | 204 | 2 |
| 38 | Raffinose | Sugar | 20.62 | 3502.15 | 361 | 2 |
| 39 | Sorbose | Sugar | 12.20 | 1905.36 | 103 | 2 |
| 40 | Sucrose | Sugar | 16.79 | 2705.66 | 361 | 2 |
| 41 | Tagatose | Sugar | 12.04 | 1883.11 | 103 | 2 |
| 42 | Xylulose+Ribulose | Sugar | 10.74 | 1701.96 | 147 | 2 |
| 43 | Arabitol | Sugar Alcohol | 11.08 | 1747.16 | 217 | 2 |
| 44 | Galactitol | Sugar Alcohol | 12.68 | 1977.08 | 217 | 2 |
| 45 | Inositol | Sugar Alcohol | 13.66 | 2132.34 | 305 | 2 |
| 46 | Mannitol | Sugar Alcohol | 12.62 | 1968.42 | 147 | 2 |
| 47 | Pinitol | Sugar Alcohol | 13.08 | 2038.98 | 217 | 2 |
| 48 | Sorbitol | Sugar Alcohol | 12.67 | 1975.49 | 147 | 2 |
| 49 | Xylitol | Sugar Alcohol | 10.99 | 1735.01 | 217 | 2 |
| 50 | 1,6-Anhydroglucose | Others | 10.92 | 1726.23 | 204 | 2 |
| 51 | 2-Hydroxybutyrate | Others | 5.57 | 1133.56 | 131 | 2 |
| 52 | 2-Hydroxypyridine | Others | 4.52 | 1039.09 | 152 | 2 |
| 53 | 3,4-Dihydroxybenzoate | Others | 6.91 | 1260.39 | 103 | 2 |
| 54 | 3-Hydroxybenzoate | Others | 9.73 | 1573.10 | 223 | 2 |
| 55 | Adenine | Others | 12.05 | 1884.25 | 264 | 2 |
| 56 | Caffeine | Others | 11.92 | 1865.62 | 194 | 2 |
| 57 | Catechol | Others | 7.58 | 1327.60 | 254 | 2 |
| 58 | Glucono-1,5-lactone | Others | 12.33 | 1925.39 | 204 | 2 |
| 59 | Glycerol | Others | 7.15 | 1283.37 | 147 | 2 |
| 60 | N-Methylnicotinate | Others | 9.26 | 1514.87 | 210 | 2 |
| 61 | Oxalacetic acid+Pyruvate | Others | 4.66 | 1051.89 | 174 | 2 |
| 62 | Phosphate | Others | 7.14 | 1282.73 | 299 | 2 |
| 63 | Pyrogallol | Others | 9.63 | 1560.20 | 239 | 2 |
| 64 | Unknown_1 | Unknown | 4.06 | 998.24 | 155 | 4 |
| 65 | Unknown_2 | Unknown | 4.61 | 1046.93 | 117 | 4 |
| 66 | Unknown_3 | Unknown | 5.73 | 1147.94 | 142 | 4 |
| 67 | Unknown_4 | Unknown | 5.74 | 1148.57 | 177 | 4 |
| 68 | Unknown_5 | Unknown | 6.20 | 1191.14 | 166 | 4 |
| 69 | Unknown_6 | Unknown | 6.33 | 1202.83 | 166 | 4 |
| 70 | Unknown_7 | Unknown | 6.51 | 1220.77 | 117 | 4 |
| 71 | Unknown_8 | Unknown | 6.96 | 1264.88 | 98 | 4 |
| 72 | Unknown_9 | Unknown | 7.25 | 1293.16 | 183 | 4 |
| 73 | Unknown_10 | Unknown | 8.27 | 1401.35 | 184 | 4 |
| 74 | Unknown_11 | Unknown | 8.29 | 1403.61 | 147 | 4 |
| 75 | Unknown_12 | Unknown | 8.31 | 1405.48 | 138 | 4 |
| 76 | Unknown_13 | Unknown | 8.36 | 1412.10 | 100 | 4 |
| 77 | Unknown_14 | Unknown | 8.39 | 1415.07 | 217 | 4 |
| 78 | Unknown_15 | Unknown | 8.45 | 1421.80 | 169 | 4 |
| 79 | Unknown_16 | Unknown | 8.46 | 1422.61 | 103 | 4 |
| 80 | Unknown_17 | Unknown | 9.10 | 1496.23 | 271 | 4 |
| 81 | Unknown_18 | Unknown | 9.15 | 1502.25 | 254 | 4 |
| 82 | Unknown_19 | Unknown | 9.21 | 1509.61 | 117 | 4 |
| 83 | Unknown_20 | Unknown | 9.28 | 1517.33 | 217 | 4 |
| 84 | Unknown_21 | Unknown | 9.28 | 1517.90 | 143 | 4 |
| 85 | Unknown_22 | Unknown | 9.28 | 1517.91 | 147 | 4 |
| 86 | Unknown_23 | Unknown | 9.29 | 1519.59 | 97 | 4 |
| 87 | Unknown_24 | Unknown | 9.37 | 1528.68 | 117 | 4 |
| 88 | Unknown_25 | Unknown | 9.49 | 1543.98 | 254 | 4 |
| 89 | Unknown_26 | Unknown | 9.60 | 1556.50 | 147 | 4 |
| 90 | Unknown_27 | Unknown | 9.91 | 1594.04 | 210 | 4 |
| 91 | Unknown_28 | Unknown | 10.08 | 1615.90 | 239 | 4 |
| 92 | Unknown_29 | Unknown | 10.11 | 1620.45 | 217 | 4 |
| 93 | Unknown_30 | Unknown | 10.23 | 1635.47 | 147 | 4 |
| 94 | Unknown_31 | Unknown | 10.47 | 1666.54 | 245 | 4 |
| 95 | Unknown_32 | Unknown | 10.59 | 1682.45 | 160 | 4 |
| 96 | Unknown_33 | Unknown | 10.59 | 1682.45 | 264 | 4 |
| 97 | Unknown_34 | Unknown | 11.00 | 1736.10 | 103 | 4 |
| 98 | Unknown_35 | Unknown | 11.07 | 1745.88 | 117 | 4 |
| 99 | Unknown_36 | Unknown | 11.26 | 1772.70 | 147 | 4 |
| 100 | Unknown_37 | Unknown | 11.30 | 1777.76 | 169 | 4 |
| 101 | Unknown_38 | Unknown | 11.31 | 1778.35 | 231 | 4 |
| 102 | Unknown_39 | Unknown | 11.39 | 1789.91 | 147 | 4 |
| 103 | Unknown_40 | Unknown | 11.42 | 1794.27 | 147 | 4 |
| 104 | Unknown_41 | Unknown | 11.46 | 1799.83 | 334 | 4 |
| 105 | Unknown_42 | Unknown | 11.46 | 1800.01 | 129 | 4 |
| 106 | Unknown_43 | Unknown | 11.53 | 1810.05 | 147 | 4 |
| 107 | Unknown_44 | Unknown | 11.54 | 1810.17 | 129 | 4 |
| 108 | Unknown_45 | Unknown | 11.57 | 1814.52 | 204 | 4 |
| 109 | Unknown_46 | Unknown | 11.72 | 1836.96 | 204 | 4 |
| 110 | Unknown_47 | Unknown | 12.01 | 1877.80 | 245 | 4 |
| 111 | Unknown_48 | Unknown | 12.04 | 1881.90 | 245 | 4 |
| 112 | Unknown_49 | Unknown | 12.08 | 1887.54 | 245 | 4 |
| 113 | Unknown_50 | Unknown | 12.09 | 1889.77 | 217 | 4 |
| 114 | Unknown_51 | Unknown | 12.11 | 1891.92 | 255 | 4 |
| 115 | Unknown_52 | Unknown | 12.56 | 1960.26 | 345 | 4 |
| 116 | Unknown_53 | Unknown | 12.58 | 1962.19 | 179 | 4 |
| 117 | Unknown_54 | Unknown | 12.59 | 1964.78 | 204 | 4 |
| 118 | Unknown_55 | Unknown | 12.66 | 1974.06 | 345 | 4 |
| 119 | Unknown_56 | Unknown | 14.16 | 2214.55 | 204 | 4 |
| 120 | Unknown_57 | Unknown | 14.24 | 2228.50 | 204 | 4 |
| 121 | Unknown_58 | Unknown | 15.27 | 2411.07 | 217 | 4 |
| 122 | Unknown_59 | Unknown | 16.15 | 2577.23 | 120 | 4 |
| 123 | Unknown_60 | Unknown | 16.26 | 2597.95 | 371 | 4 |
| 124 | Unknown_61 | Unknown | 16.64 | 2675.38 | 217 | 4 |
| 125 | Unknown_62 | Unknown | 16.65 | 2677.06 | 169 | 4 |
| 126 | Unknown_63 | Unknown | 16.66 | 2678.32 | 217 | 4 |
| 127 | Unknown_64 | Unknown | 17.05 | 2757.70 | 271 | 4 |
| 128 | Unknown_65 | Unknown | 17.11 | 2771.39 | 120 | 4 |
| 129 | Unknown_66 | Unknown | 17.15 | 2778.82 | 217 | 4 |
| 130 | Unknown_67 | Unknown | 18.01 | 2965.55 | 340 | 4 |
| 131 | Unknown_68 | Unknown | 18.20 | 3009.43 | 193 | 4 |
| 132 | Unknown_69 | Unknown | 18.80 | 3146.28 | 345 | 4 |
| 133 | Unknown_70 | Unknown | 18.91 | 3171.09 | 255 | 4 |
| 134 | Unknown_71 | Unknown | 19.13 | 3220.59 | 307 | 4 |

There are 134 metabolites detected in all brewed coffee samples

a Retention time in minute(s)

b Retention indices (RI) are calculated using a standard alkane mixture (C10–C40).

c The MSI confidence level of annotation and identification. Mass spectra of metabolite peaks that are compared with the library and the authentic standard considered as Metabolic Standards Initiative (MSI) level 1. Mass spectra of metabolite peaks only with the in-house library are considered MSI level 2 for putatively annotated compound and MSI level 3 for putatively characterized compound classes. The metabolite peak categorized as MSI level 4 if there are no similarities with any chemical class compound in library (Sumner et al. 2007).

Table S5 Evaluation of the models obtained from OPLS-R analysis

| y variable | A | N | R^2^X(cum) | R^2^Y(cum) | Q^2^(cum) | *p*-value |
| --- | --- | --- | --- | --- | --- | --- |
| Sample set 1 |  |  |  |  |  |  |
| Final Score - Green Bean | 1+4+0 | 30 | 0.683 | 0.993 | 0.978 | 1.66E-13 |
| Final Score - Roasted Bean | 1+4+0 | 30 | 0.741 | 0.996 | 0.97 | 3.01E-12 |
| Final Score - Brewed Coffee | 1+4+0 | 30 | 0.735 | 0.986 | 0.93 | 7.39E-09 |
| Sample set 2 |  |  |  |  |  |  |
| Final Score Set - Green Bean | 1+3+0 | 105 | 0.348 | 0.93 | 0.885 | 0 |

A, number of models. N, number of samples used in producing the models. Cross-validated predictive residuals (CV-ANOVA) analysis was employed to obtain *p*-values.

Table S6 List of metabolites sorted based on VIP value in green coffee bean “Final Score” model

| No | Metabolite Name | VIP | Coefficient |
| --- | --- | --- | --- |
| 1 | Unknown_45 | 2.156 | 0.0530 |
| 2 | Galactinol | 2.008 | 0.0533 |
| 3 | Unknown_34 | 1.978 | -0.0649 |
| 4 | Unknown_18 | 1.943 | 0.0562 |
| 5 | Shikimic acid | 1.713 | -0.0656 |
| 6 | Glutamic acid | 1.701 | 0.0203 |
| 7 | Caffeine | 1.660 | 0.0408 |
| 8 | Unknown_2 | 1.613 | 0.0918 |
| 9 | Trehalose | 1.613 | 0.0415 |
| 10 | Unknown_15 | 1.544 | -0.0514 |
| 11 | Unknown_16 | 1.538 | 0.0695 |
| 12 | Alanine | 1.515 | -0.0390 |
| 13 | Ferulic acid | 1.472 | -0.0362 |
| 14 | Unknown_13 | 1.451 | -0.0129 |
| 15 | Unknown_8 | 1.446 | -0.0103 |
| 16 | N-Methylnicotinate | 1.443 | -0.0145 |
| 17 | Glycerol | 1.435 | -0.0377 |
| 18 | Unknown_9 | 1.428 | 0.0271 |
| 19 | Unknown_1 | 1.424 | 0.0907 |
| 20 | Threitol | 1.421 | -0.0497 |
| 21 | Melezitose | 1.395 | 0.0277 |
| 22 | Unknown_55 | 1.375 | 0.0386 |
| 23 | b-Glutamic acid | 1.372 | -0.0219 |
| 24 | Unknown_56 | 1.353 | -0.0241 |
| 25 | Unknown_3 | 1.351 | -0.0363 |
| 26 | Unknown_54 | 1.304 | 0.0358 |
| 27 | Glutamine | 1.298 | 0.0176 |
| 28 | Unknown_32 | 1.297 | -0.0004 |
| 29 | Mannose | 1.295 | -0.0131 |
| 30 | Unknown_43 | 1.258 | 0.0219 |
| 31 | Unknown_49 | 1.258 | -0.0358 |
| 32 | Glycolic acid | 1.234 | 0.0427 |
| 33 | Sorbitol | 1.208 | -0.0269 |
| 34 | Inositol | 1.207 | -0.0009 |
| 35 | Unknown_35 | 1.195 | 0.0057 |
| 36 | Unknown_36 | 1.189 | -0.0025 |
| 37 | Gentiobiose | 1.177 | -0.0186 |
| 38 | 2-Dehydro gluconate | 1.170 | 0.0230 |
| 39 | Phenylalanine | 1.159 | 0.0516 |
| 40 | Unknown_17 | 1.155 | -0.0080 |
| 41 | Paeoniflorin | 1.151 | -0.0572 |
| 42 | Glycine | 1.146 | -0.0172 |
| 43 | Unknown_20 | 1.130 | -0.0183 |
| 44 | Galacturonic acid | 1.128 | -0.0125 |
| 45 | Unknown_23 | 1.126 | -0.0283 |
| 46 | Phosphate | 1.125 | -0.0096 |
| 47 | Sucrose | 1.105 | 0.0411 |
| 48 | Unknown_21 | 1.097 | -0.0108 |
| 49 | Tryptophan | 1.090 | 0.0019 |
| 50 | Unknown_50 | 1.089 | -0.0132 |
| 51 | Unknown_47 | 1.064 | -0.0136 |
| 52 | Xylitol | 1.056 | -0.0018 |
| 53 | Meso erythritol | 1.054 | -0.0132 |
| 54 | Unknown_12 | 1.053 | -0.0087 |
| 55 | Glyceric acid | 1.046 | 0.0033 |
| 56 | Fumaric acid | 1.035 | -0.0067 |
| 57 | Unknown_52 | 0.954 | 0.0443 |
| 58 | Galactose | 0.909 | -0.0064 |
| 59 | Succinic acid | 0.906 | -0.0241 |
| 60 | Methyl succinic acid | 0.874 | -0.0388 |
| 61 | Unknown_39 | 0.871 | -0.0038 |
| 62 | Unknown_7 | 0.868 | -0.0114 |
| 63 | Lactic acid | 0.835 | 0.0002 |
| 64 | Unknown_30 | 0.832 | -0.0243 |
| 65 | Unknown_40 | 0.813 | -0.0140 |
| 66 | Unknown_37 | 0.787 | -0.0003 |
| 67 | Unknown_48 | 0.776 | -0.0034 |
| 68 | Unknown_19 | 0.769 | -0.0186 |
| 69 | Unknown_53 | 0.769 | -0.0068 |
| 70 | Unknown_27 | 0.751 | -0.0020 |
| 71 | Unknown_10 | 0.749 | 0.0495 |
| 72 | Xylonic acid | 0.699 | 0.0063 |
| 73 | Proline_2TMS | 0.685 | -0.0106 |
| 74 | Unknown_5 | 0.658 | 0.0187 |
| 75 | Unknown_11 | 0.656 | 0.0477 |
| 76 | Unknown_29 | 0.646 | 0.0214 |
| 77 | Raffinose | 0.635 | 0.0024 |
| 78 | Aspartic acid | 0.625 | -0.0081 |
| 79 | Putrescine | 0.623 | 0.0442 |
| 80 | Mannitol | 0.590 | -0.0468 |
| 81 | Nicotinic acid | 0.588 | 0.0037 |
| 82 | Galactose+Glucose | 0.585 | -0.0246 |
| 83 | Glucose | 0.573 | -0.0228 |
| 84 | Glucarate | 0.572 | 0.0105 |
| 85 | Unknown_46 | 0.552 | 0.0055 |
| 86 | Unknown_31 | 0.528 | -0.0039 |
| 87 | Serine | 0.516 | -0.0306 |
| 88 | Unknown_41 | 0.505 | 0.0002 |
| 89 | Valine | 0.494 | 0.0054 |
| 90 | Glucono-1,5-lactone | 0.480 | -0.0012 |
| 91 | Threonine | 0.475 | -0.0016 |
| 92 | 2-Aminoethanol | 0.475 | 0.0249 |
| 93 | Gluconic acid | 0.412 | 0.0017 |
| 94 | Unknown_33 | 0.401 | 0.0100 |
| 95 | Unknown_26 | 0.378 | -0.0011 |
| 96 | Unknown_24 | 0.360 | 0.0477 |
| 97 | Pyroglutamic acid | 0.360 | -0.0356 |
| 98 | Unknown_28 | 0.340 | 0.0298 |
| 99 | Tyrosine | 0.317 | 0.0174 |
| 100 | Lysine | 0.299 | 0.0153 |
| 101 | Unknown_4 | 0.283 | 0.0000 |
| 102 | Citric acid | 0.254 | 0.0289 |
| 103 | Unknown_22 | 0.243 | 0.0376 |
| 104 | Isoleucine | 0.227 | 0.0173 |
| 105 | Galactitol | 0.198 | -0.0214 |
| 106 | Malic acid | 0.181 | -0.0423 |
| 107 | Quinic acid | 0.154 | 0.0012 |
| 108 | Chlorogenic acid | 0.146 | 0.0062 |
| 109 | 4-Aminobutyric acid | 0.137 | 0.0379 |
| 110 | Unknown_38 | 0.127 | -0.0329 |
| 111 | Fructose | 0.107 | 0.0001 |
| 112 | Unknown_14 | 0.104 | -0.0123 |
| 113 | Psicose+Tagatose | 0.092 | 0.0001 |
| 114 | Sorbose | 0.091 | 0.0001 |
| 115 | Unknown_6 | 0.085 | -0.0104 |
| 116 | Unknown_42 | 0.084 | -0.0105 |
| 117 | Leucine | 0.080 | 0.0254 |
| 118 | Unknown_51 | 0.073 | 0.0012 |
| 119 | Panose | 0.039 | 0.0229 |
| 120 | Asparagine | 0.024 | -0.0074 |
| 121 | Unknown_44 | 0.021 | -0.0346 |
| 122 | Serotonin | 0.017 | -0.0086 |
| 123 | Unknown_25 | 0.015 | -0.0128 |
| 124 | Adenosine | 0.012 | 0.0182 |

Table S7 List of metabolites sorted based on VIP value in roasted coffee bean “Final Score” model

| No | Metabolite Name | VIP | Coefficient |
| --- | --- | --- | --- |
| 1 | Unknown_191 | 1.849 | 0.0283 |
| 2 | Unknown_101 | 1.823 | 0.0257 |
| 3 | Unknown_55 | 1.794 | 0.0220 |
| 4 | Unknown_54 | 1.761 | 0.0196 |
| 5 | Galactinol | 1.759 | 0.0215 |
| 6 | Unknown_128 | 1.757 | 0.0203 |
| 7 | Fructose | 1.747 | 0.0195 |
| 8 | Galactose+Glucose | 1.744 | 0.0185 |
| 9 | Psicose+Tagatose | 1.742 | 0.0195 |
| 10 | Glucose | 1.742 | 0.0191 |
| 11 | Allose+Mannose | 1.741 | 0.0272 |
| 12 | Galactose | 1.741 | 0.0232 |
| 13 | Tagatose | 1.740 | 0.0189 |
| 14 | Mannose | 1.739 | 0.0192 |
| 15 | Unknown_124 | 1.737 | 0.0185 |
| 16 | Unknown_99 | 1.725 | 0.0169 |
| 17 | Allose | 1.725 | 0.0169 |
| 18 | Dihydroxyacetone | 1.723 | 0.0192 |
| 19 | Unknown_125 | 1.712 | 0.0154 |
| 20 | Unknown_135 | 1.702 | 0.0171 |
| 21 | Turanose | 1.641 | 0.0145 |
| 22 | Unknown_20 | 1.635 | 0.0122 |
| 23 | Unknown_203 | 1.602 | 0.0116 |
| 24 | Unknown_88 | 1.594 | 0.0153 |
| 25 | Unknown_82 | 1.594 | 0.0117 |
| 26 | Unknown_67 | 1.584 | 0.0146 |
| 27 | Unknown_81 | 1.584 | 0.0118 |
| 28 | Fumaric acid | 1.583 | -0.0240 |
| 29 | Unknown_175 | 1.560 | 0.0075 |
| 30 | Psicose | 1.542 | 0.0175 |
| 31 | Unknown_46 | 1.538 | 0.0141 |
| 32 | Unknown_12 | 1.499 | 0.0097 |
| 33 | Unknown_48 | 1.491 | 0.0080 |
| 34 | Unknown_6 | 1.480 | 0.0094 |
| 35 | Unknown_37 | 1.477 | 0.0094 |
| 36 | Unknown_201 | 1.467 | 0.0082 |
| 37 | Unknown_169 | 1.463 | 0.0123 |
| 38 | Unknown_7 | 1.441 | -0.0072 |
| 39 | Unknown_122 | 1.439 | 0.0086 |
| 40 | Unknown_119 | 1.410 | 0.0228 |
| 41 | Unknown_172 | 1.404 | 0.0116 |
| 42 | Unknown_63 | 1.399 | 0.0106 |
| 43 | Unknown_202 | 1.395 | 0.0049 |
| 44 | Unknown_100 | 1.394 | 0.0009 |
| 45 | Unknown_76 | 1.382 | 0.0040 |
| 46 | Unknown_74 | 1.382 | 0.0161 |
| 47 | Lyxose | 1.378 | 0.0113 |
| 48 | Unknown_69 | 1.369 | 0.0068 |
| 49 | Unknown_85 | 1.358 | 0.0102 |
| 50 | Unknown_171 | 1.356 | 0.0053 |
| 51 | Unknown_165 | 1.341 | 0.0048 |
| 52 | Unknown_126 | 1.340 | 0.0177 |
| 53 | Unknown_168 | 1.337 | 0.0088 |
| 54 | Maltose | 1.306 | 0.0087 |
| 55 | Cytosine | 1.304 | -0.0006 |
| 56 | Ribose | 1.303 | 0.0102 |
| 57 | Xylulose+Ribulose | 1.301 | 0.0101 |
| 58 | Unknown_198 | 1.300 | 0.0084 |
| 59 | Unknown_167 | 1.295 | 0.0139 |
| 60 | Unknown_72 | 1.295 | 0.0012 |
| 61 | Unknown_176 | 1.291 | 0.0026 |
| 62 | Arabionose | 1.291 | 0.0179 |
| 63 | Unknown_26 | 1.278 | -0.0350 |
| 64 | Melezitose | 1.265 | 0.0152 |
| 65 | Trehalose | 1.262 | 0.0148 |
| 66 | Sucrose | 1.215 | 0.0016 |
| 67 | Unknown_195 | 1.200 | 0.0061 |
| 68 | Unknown_28 | 1.193 | -0.0192 |
| 69 | Unknown_196 | 1.190 | 0.0124 |
| 70 | Unknown_157 | 1.185 | -0.0031 |
| 71 | Unknown_75 | 1.177 | 0.0009 |
| 72 | Unknown_49 | 1.165 | 0.0015 |
| 73 | Unknown_41 | 1.154 | 0.0023 |
| 74 | Glycerol | 1.152 | -0.0359 |
| 75 | Unknown_42 | 1.141 | 0.0025 |
| 76 | Unknown_86 | 1.141 | 0.0082 |
| 77 | Unknown_8 | 1.137 | 0.0039 |
| 78 | Unknown_25 | 1.136 | -0.0280 |
| 79 | Inositol | 1.131 | -0.0187 |
| 80 | Unknown_83 | 1.126 | -0.0026 |
| 81 | Phosphate | 1.119 | -0.0259 |
| 82 | Unknown_131 | 1.109 | -0.0158 |
| 83 | Melibiose | 1.108 | 0.0041 |
| 84 | Adenine | 1.107 | -0.0088 |
| 85 | Unknown_145 | 1.106 | 0.0264 |
| 86 | Unknown_40 | 1.102 | 0.0090 |
| 87 | Unknown_182 | 1.097 | -0.0143 |
| 88 | Unknown_104 | 1.089 | 0.0024 |
| 89 | Unknown_105 | 1.089 | 0.0033 |
| 90 | Unknown_127 | 1.085 | -0.0094 |
| 91 | Unknown_151 | 1.083 | -0.0029 |
| 92 | Unknown_66 | 1.077 | 0.0072 |
| 93 | Unknown_164 | 1.069 | -0.0032 |
| 94 | Chlorogenic acid | 1.066 | -0.0019 |
| 95 | Unknown_193 | 1.065 | -0.0093 |
| 96 | Unknown_56 | 1.058 | -0.0020 |
| 97 | Unknown_52 | 1.057 | -0.0003 |
| 98 | Unknown_27 | 1.050 | 0.0021 |
| 99 | Unknown_194 | 1.048 | -0.0101 |
| 100 | Unknown_32 | 1.045 | -0.0030 |
| 101 | 2-Hydroxybutyrate | 1.043 | 0.0055 |
| 102 | Unknown_173 | 1.040 | 0.0087 |
| 103 | Unknown_192 | 1.036 | -0.0086 |
| 104 | Propyleneglycol | 1.034 | -0.0143 |
| 105 | Unknown_51 | 1.024 | -0.0155 |
| 106 | Unknown_22 | 1.021 | -0.0165 |
| 107 | Unknown_186 | 1.017 | -0.0126 |
| 108 | Methyl succinic acid | 1.017 | 0.0005 |
| 109 | Unknown_185 | 1.013 | -0.0177 |
| 110 | Unknown_65 | 1.013 | 0.0032 |
| 111 | Lactic acid | 1.009 | 0.0058 |
| 112 | Unknown_181 | 1.003 | 0.0007 |
| 113 | Meso erythritol | 1.002 | -0.0399 |
| 114 | Unknown_120 | 0.992 | -0.0092 |
| 115 | Unknown_13 | 0.991 | 0.0068 |
| 116 | Unknown_136 | 0.980 | 0.0009 |
| 117 | Unknown_102 | 0.978 | -0.0171 |
| 118 | Unknown_18 | 0.976 | -0.0023 |
| 119 | Unknown_143 | 0.973 | -0.0230 |
| 120 | Unknown_59 | 0.967 | 0.0038 |
| 121 | Unknown_61 | 0.964 | -0.0055 |
| 122 | Unknown_60 | 0.963 | 0.0049 |
| 123 | Unknown_179 | 0.956 | 0.0009 |
| 124 | Oxalacetic acid+Pyruvate | 0.955 | -0.0019 |
| 125 | Unknown_114 | 0.945 | 0.0002 |
| 126 | Unknown_44 | 0.944 | -0.0023 |
| 127 | Unknown_106 | 0.935 | 0.0033 |
| 128 | Unknown_180 | 0.931 | 0.0036 |
| 129 | Unknown_80 | 0.928 | 0.0189 |
| 130 | Unknown_132 | 0.928 | 0.0206 |
| 131 | Shikimic acid | 0.927 | 0.0004 |
| 132 | Pyrogallol | 0.915 | 0.0047 |
| 133 | Quinic acid | 0.912 | -0.0091 |
| 134 | Unknown_33 | 0.910 | 0.0041 |
| 135 | Unknown_96 | 0.908 | -0.0014 |
| 136 | Unknown_197 | 0.906 | 0.0030 |
| 137 | Unknown_159 | 0.906 | -0.0025 |
| 138 | Unknown_17 | 0.906 | 0.0045 |
| 139 | Caffeine | 0.903 | 0.0418 |
| 140 | Threonic acid | 0.901 | -0.0041 |
| 141 | Unknown_121 | 0.899 | 0.0001 |
| 142 | Catechol | 0.898 | 0.0011 |
| 143 | Rhamnose | 0.890 | 0.0151 |
| 144 | Unknown_177 | 0.877 | 0.0091 |
| 145 | Unknown_204 | 0.874 | -0.0023 |
| 146 | Unknown_107 | 0.869 | 0.0024 |
| 147 | Unknown_200 | 0.863 | -0.0035 |
| 148 | Unknown_43 | 0.862 | 0.0062 |
| 149 | Unknown_4 | 0.861 | 0.0050 |
| 150 | 4-Aminobutyric acid | 0.856 | 0.0032 |
| 151 | Unknown_15 | 0.853 | 0.0031 |
| 152 | Unknown_130 | 0.852 | 0.0044 |
| 153 | Unknown_152 | 0.850 | 0.0094 |
| 154 | Unknown_154 | 0.842 | 0.0110 |
| 155 | Succinic acid | 0.828 | 0.0008 |
| 156 | b-Lactose | 0.827 | 0.0020 |
| 157 | Unknown_112 | 0.815 | -0.0010 |
| 158 | Unknown_68 | 0.812 | -0.0240 |
| 159 | Sorbitol | 0.802 | -0.0165 |
| 160 | Unknown_188 | 0.801 | 0.0033 |
| 161 | Unknown_71 | 0.798 | 0.0031 |
| 162 | Unknown_133 | 0.797 | -0.0005 |
| 163 | Unknown_116 | 0.794 | 0.0015 |
| 164 | Lauric acid | 0.793 | -0.0062 |
| 165 | Unknown_153 | 0.790 | 0.0008 |
| 166 | Unknown_109 | 0.790 | 0.0018 |
| 167 | Unknown_31 | 0.788 | 0.0063 |
| 168 | 3-Hydroxybenzoate | 0.786 | 0.0037 |
| 169 | Unknown_115 | 0.779 | 0.0015 |
| 170 | Unknown_129 | 0.777 | 0.0055 |
| 171 | Xylitol | 0.771 | -0.0094 |
| 172 | Unknown_45 | 0.767 | 0.0053 |
| 173 | Unknown_57 | 0.763 | -0.0038 |
| 174 | Unknown_98 | 0.755 | -0.0063 |
| 175 | Unknown_118 | 0.753 | 0.0021 |
| 176 | Unknown_162 | 0.751 | 0.0031 |
| 177 | Unknown_36 | 0.735 | -0.0060 |
| 178 | Unknown_97 | 0.733 | 0.0054 |
| 179 | Unknown_87 | 0.717 | 0.0047 |
| 180 | Unknown_35 | 0.713 | -0.0051 |
| 181 | Unknown_62 | 0.710 | 0.0049 |
| 182 | Unknown_160 | 0.704 | -0.0050 |
| 183 | Unknown_117 | 0.703 | 0.0058 |
| 184 | Unknown_110 | 0.702 | 0.0076 |
| 185 | Unknown_38 | 0.693 | 0.0041 |
| 186 | Unknown_144 | 0.688 | -0.0029 |
| 187 | Unknown_3 | 0.688 | 0.0070 |
| 188 | Unknown_184 | 0.672 | -0.0287 |
| 189 | Unknown_178 | 0.670 | -0.0140 |
| 190 | Unknown_150 | 0.668 | 0.0296 |
| 191 | Unknown_155 | 0.652 | -0.0212 |
| 192 | 3,4-Dihydroxybenzoate | 0.649 | -0.0083 |
| 193 | Unknown_123 | 0.645 | -0.0050 |
| 194 | Unknown_23 | 0.636 | -0.0066 |
| 195 | Unknown_111 | 0.607 | -0.0011 |
| 196 | Unknown_95 | 0.603 | -0.0017 |
| 197 | Unknown_79 | 0.594 | -0.0042 |
| 198 | Unknown_84 | 0.588 | 0.0043 |
| 199 | Unknown_94 | 0.584 | -0.0073 |
| 200 | Nicotinic acid | 0.582 | 0.0011 |
| 201 | Unknown_149 | 0.579 | 0.0001 |
| 202 | Malic acid | 0.576 | -0.0207 |
| 203 | Unknown_166 | 0.574 | -0.0068 |
| 204 | Unknown_140 | 0.561 | 0.0181 |
| 205 | Unknown_2 | 0.560 | 0.0264 |
| 206 | Unknown_30 | 0.559 | -0.0039 |
| 207 | Glucono-1,5-lactone | 0.555 | 0.0085 |
| 208 | Unknown_137 | 0.553 | -0.0268 |
| 209 | Unknown_108 | 0.549 | 0.0222 |
| 210 | Unknown_91 | 0.534 | -0.0196 |
| 211 | Unknown_207 | 0.525 | 0.0352 |
| 212 | Unknown_158 | 0.523 | -0.0040 |
| 213 | Unknown_148 | 0.519 | 0.0075 |
| 214 | Unknown_190 | 0.517 | 0.0144 |
| 215 | Unknown_146 | 0.517 | 0.0140 |
| 216 | Unknown_147 | 0.507 | 0.0164 |
| 217 | Unknown_170 | 0.506 | -0.0149 |
| 218 | Unknown_174 | 0.491 | 0.0026 |
| 219 | Mannitol | 0.488 | -0.0383 |
| 220 | Unknown_163 | 0.463 | 0.0097 |
| 221 | Unknown_19 | 0.457 | -0.0014 |
| 222 | Panose | 0.447 | 0.0324 |
| 223 | Unknown_11 | 0.446 | -0.0071 |
| 224 | Unknown_205 | 0.440 | 0.0326 |
| 225 | Unknown_47 | 0.439 | 0.0097 |
| 226 | Unknown_183 | 0.434 | 0.0069 |
| 227 | Glycolic acid | 0.428 | 0.0038 |
| 228 | Unknown_113 | 0.418 | 0.0441 |
| 229 | Unknown_21 | 0.407 | 0.0014 |
| 230 | Stearic acid | 0.404 | 0.0027 |
| 231 | Gluconic acid | 0.398 | -0.0046 |
| 232 | Unknown_199 | 0.395 | -0.0028 |
| 233 | Unknown_206 | 0.378 | 0.0297 |
| 234 | Citric acid | 0.375 | 0.0432 |
| 235 | Unknown_78 | 0.366 | -0.0087 |
| 236 | Unknown_9 | 0.365 | 0.0103 |
| 237 | Unknown_1 | 0.361 | 0.0006 |
| 238 | Plamitic acid | 0.335 | 0.0038 |
| 239 | Unknown_139 | 0.312 | 0.0248 |
| 240 | Unknown_5 | 0.309 | -0.0103 |
| 241 | 5-Oxoproline | 0.307 | 0.0182 |
| 242 | Unknown_64 | 0.304 | -0.0007 |
| 243 | Unknown_138 | 0.295 | -0.0093 |
| 244 | Unknown_34 | 0.292 | -0.0050 |
| 245 | Unknown_70 | 0.292 | 0.0104 |
| 246 | Maltitol | 0.291 | -0.0133 |
| 247 | 2-Hydroxypyridine | 0.269 | 0.0058 |
| 248 | Unknown_141 | 0.264 | 0.0014 |
| 249 | Unknown_103 | 0.255 | 0.0115 |
| 250 | Unknown_89 | 0.247 | -0.0181 |
| 251 | Unknown_93 | 0.246 | -0.0267 |
| 252 | Unknown_50 | 0.225 | -0.0112 |
| 253 | Unknown_187 | 0.200 | -0.0209 |
| 254 | Unknown_53 | 0.199 | -0.0078 |
| 255 | Unknown_161 | 0.197 | 0.0090 |
| 256 | Arabitol | 0.190 | -0.0129 |
| 257 | Unknown_156 | 0.185 | -0.0154 |
| 258 | N-Methylnicotinate | 0.178 | -0.0098 |
| 259 | Glucarate_2_same | 0.170 | -0.0175 |
| 260 | Maleic acid | 0.163 | -0.0126 |
| 261 | Unknown_16 | 0.151 | -0.0025 |
| 262 | Galacturonic acid | 0.150 | -0.0170 |
| 263 | Unknown_39 | 0.131 | 0.0032 |
| 264 | Unknown_58 | 0.127 | 0.0038 |
| 265 | Aspartic acid_3TMS | 0.127 | 0.0155 |
| 266 | Unknown_77 | 0.127 | -0.0018 |
| 267 | Glyceric acid | 0.119 | -0.0080 |
| 268 | 1,6-Anhydroglucose | 0.115 | 0.0179 |
| 269 | Unknown_29 | 0.115 | -0.0182 |
| 270 | Unknown_90 | 0.089 | -0.0104 |
| 271 | Myristic acid | 0.085 | 0.0105 |
| 272 | Unknown_73 | 0.062 | -0.0160 |
| 273 | Unknown_92 | 0.058 | 0.0057 |
| 274 | Unknown_134 | 0.050 | -0.0268 |
| 275 | Unknown_14 | 0.049 | -0.0132 |
| 276 | Unknown_189 | 0.043 | -0.0244 |
| 277 | Unknown_10 | 0.035 | -0.0041 |
| 278 | Unknown_142 | 0.024 | -0.0195 |
| 279 | Unknown_24 | 0.022 | 0.0323 |

Table S8 List of metabolites sorted based on VIP value in brewed coffee “Final Score” model

| No | Metabolites Name | VIP | Coefficient |
| --- | --- | --- | --- |
| 1 | Unknown_50 | 2.018 | 0.0610 |
| 2 | Mannose | 1.809 | 0.0325 |
| 3 | Fructose | 1.782 | 0.0320 |
| 4 | Galactose+Glucose | 1.774 | 0.0311 |
| 5 | Unknown_38 | 1.767 | 0.0326 |
| 6 | Sorbose | 1.766 | 0.0300 |
| 7 | Glucose | 1.762 | 0.0303 |
| 8 | Tagatose | 1.738 | 0.0392 |
| 9 | Allose | 1.733 | 0.0309 |
| 10 | Fumaric acid | 1.706 | -0.0390 |
| 11 | Galactinol | 1.702 | 0.0394 |
| 12 | Allose+Mannose | 1.622 | 0.0453 |
| 13 | Galactose | 1.563 | 0.0204 |
| 14 | Unknown_30 | 1.560 | 0.0237 |
| 15 | Unknown_66 | 1.541 | 0.0272 |
| 16 | Unknown_53 | 1.503 | -0.0348 |
| 17 | Arabionose | 1.499 | 0.0149 |
| 18 | Xylulose+Ribulose | 1.447 | 0.0181 |
| 19 | Sucrose | 1.435 | 0.0192 |
| 20 | Unknown_23 | 1.421 | 0.0028 |
| 21 | Raffinose | 1.420 | 0.0400 |
| 22 | Unknown_29 | 1.412 | 0.0141 |
| 23 | Unknown_15 | 1.389 | 0.0009 |
| 24 | Arabitol | 1.334 | -0.1354 |
| 25 | Unknown_42 | 1.300 | -0.0068 |
| 26 | Unknown_41 | 1.292 | -0.0061 |
| 27 | Unknown_12 | 1.289 | 0.0029 |
| 28 | Shikimic acid | 1.277 | -0.0521 |
| 29 | Sorbitol | 1.277 | -0.0413 |
| 30 | Unknown_25 | 1.255 | -0.0103 |
| 31 | Adenine | 1.247 | -0.0201 |
| 32 | Catechol | 1.244 | -0.0324 |
| 33 | Succinic acid | 1.221 | 0.0740 |
| 34 | Unknown_17 | 1.196 | -0.0050 |
| 35 | 2-Hydroxybutyrate | 1.192 | 0.0024 |
| 36 | Unknown_49 | 1.184 | 0.0090 |
| 37 | Unknown_62 | 1.174 | 0.0316 |
| 38 | Glycerol | 1.173 | -0.0664 |
| 39 | Unknown_7 | 1.167 | -0.0140 |
| 40 | Unknown_24 | 1.140 | -0.0056 |
| 41 | Unknown_20 | 1.131 | -0.0143 |
| 42 | Unknown_2 | 1.130 | 0.0509 |
| 43 | Unknown_11 | 1.120 | 0.0619 |
| 44 | Unknown_9 | 1.120 | -0.0007 |
| 45 | Unknown_37 | 1.116 | -0.0291 |
| 46 | Unknown_13 | 1.112 | -0.0200 |
| 47 | Unknown_35 | 1.106 | 0.0061 |
| 48 | Phosphate | 1.100 | -0.0321 |
| 49 | Inositol | 1.096 | -0.0247 |
| 50 | Unknown_44 | 1.081 | -0.0085 |
| 51 | Unknown_43 | 1.063 | -0.0101 |
| 52 | Unknown_19 | 1.060 | -0.0015 |
| 53 | Unknown_70 | 1.058 | 0.0054 |
| 54 | Pyrogallol | 1.049 | -0.0424 |
| 55 | Unknown_52 | 1.045 | 0.0041 |
| 56 | Unknown_6 | 1.013 | -0.0047 |
| 57 | Chlorogenic acid | 1.011 | 0.0000 |
| 58 | Unknown_5 | 1.007 | -0.0221 |
| 59 | 3,4-Dihydroxybenzoate | 0.982 | -0.0660 |
| 60 | Xylitol | 0.975 | 0.0009 |
| 61 | Unknown_55 | 0.965 | -0.0092 |
| 62 | Methyl succinic acid | 0.963 | -0.0069 |
| 63 | Maltose | 0.959 | -0.0003 |
| 64 | Unknown_45 | 0.943 | -0.0103 |
| 65 | Unknown_34 | 0.933 | 0.0031 |
| 66 | Unknown_46 | 0.917 | -0.0099 |
| 67 | Oxalacetic acid+Pyruvate | 0.911 | -0.0207 |
| 68 | Unknown_36 | 0.901 | -0.0023 |
| 69 | Maleic acid | 0.900 | -0.0033 |
| 70 | Unknown_22 | 0.872 | -0.0043 |
| 71 | Unknown_21 | 0.865 | -0.0021 |
| 72 | Unknown_4 | 0.865 | -0.0161 |
| 73 | Lactic acid | 0.846 | -0.0152 |
| 74 | Citric acid | 0.843 | 0.0360 |
| 75 | Methyl maleic acid | 0.839 | -0.0057 |
| 76 | Galactitol | 0.816 | -0.0347 |
| 77 | Unknown_48 | 0.810 | -0.0064 |
| 78 | Unknown_10 | 0.798 | -0.0037 |
| 79 | Unknown_71 | 0.790 | -0.0192 |
| 80 | Unknown_47 | 0.777 | -0.0061 |
| 81 | Unknown_27 | 0.769 | 0.0095 |
| 82 | Gluconic acid | 0.762 | 0.0084 |
| 83 | Pinitol | 0.735 | 0.0677 |
| 84 | Unknown_18 | 0.734 | 0.0005 |
| 85 | Unknown_26 | 0.689 | -0.0342 |
| 86 | N-Methylnicotinate | 0.644 | 0.0146 |
| 87 | 3-Hydroxybenzoate | 0.641 | -0.0287 |
| 88 | 2-Hydroxypyridine | 0.628 | -0.0149 |
| 89 | Unknown_14 | 0.613 | -0.0162 |
| 90 | Lyxose | 0.590 | 0.0142 |
| 91 | Unknown_58 | 0.586 | 0.0238 |
| 92 | Unknown_31 | 0.568 | 0.0021 |
| 93 | Unknown_57 | 0.526 | 0.0045 |
| 94 | Myristic acid | 0.517 | 0.0177 |
| 95 | Unknown_64 | 0.452 | 0.0370 |
| 96 | Nicotinic acid | 0.449 | 0.0130 |
| 97 | Malic acid | 0.449 | -0.0312 |
| 98 | Unknown_3 | 0.447 | 0.0214 |
| 99 | Unknown_39 | 0.439 | 0.0015 |
| 100 | Unknown_69 | 0.398 | -0.0180 |
| 101 | Quinic acid | 0.366 | 0.0188 |
| 102 | Unknown_51 | 0.347 | 0.0198 |
| 103 | Unknown_16 | 0.345 | -0.0161 |
| 104 | Glucono-1,5-lactone | 0.332 | -0.0430 |
| 105 | Unknown_1 | 0.304 | 0.0069 |
| 106 | Unknown_54 | 0.304 | -0.0835 |
| 107 | Unknown_65 | 0.286 | 0.0152 |
| 108 | Stearic acid | 0.274 | 0.0019 |
| 109 | Unknown_59 | 0.266 | 0.0113 |
| 110 | Unknown_67 | 0.263 | 0.0107 |
| 111 | Unknown_63 | 0.259 | -0.0030 |
| 112 | Oleic acid | 0.243 | -0.0100 |
| 113 | Icosanoic acid | 0.226 | -0.0087 |
| 114 | Unknown_33 | 0.225 | -0.0183 |
| 115 | Unknown_40 | 0.187 | -0.0011 |
| 116 | 9,12-Octadecadienoic acid | 0.170 | -0.0038 |
| 117 | b-Lactose | 0.165 | 0.0354 |
| 118 | Mannitol | 0.156 | -0.0950 |
| 119 | Altronic acid | 0.151 | -0.0150 |
| 120 | Glycolic acid | 0.141 | 0.0026 |
| 121 | Unknown_68 | 0.136 | -0.0479 |
| 122 | Unknown_61 | 0.100 | 0.0056 |
| 123 | Unknown_32 | 0.093 | -0.0021 |
| 124 | Galacturonic acid | 0.089 | -0.0338 |
| 125 | Unknown_56 | 0.088 | -0.0307 |
| 126 | 1,6-Anhydroglucose | 0.080 | -0.0322 |
| 127 | Glyceric acid | 0.078 | -0.0419 |
| 128 | Unknown_60 | 0.057 | -0.0128 |
| 129 | Unknown_28 | 0.051 | -0.0470 |
| 130 | Panose | 0.049 | 0.0430 |
| 131 | Pyroglutamic acid | 0.045 | 0.0313 |
| 132 | Caffeine | 0.042 | 0.0299 |
| 133 | Unknown_8 | 0.021 | 0.0426 |
| 134 | Plamitic acid | 0.011 | 0.0049 |


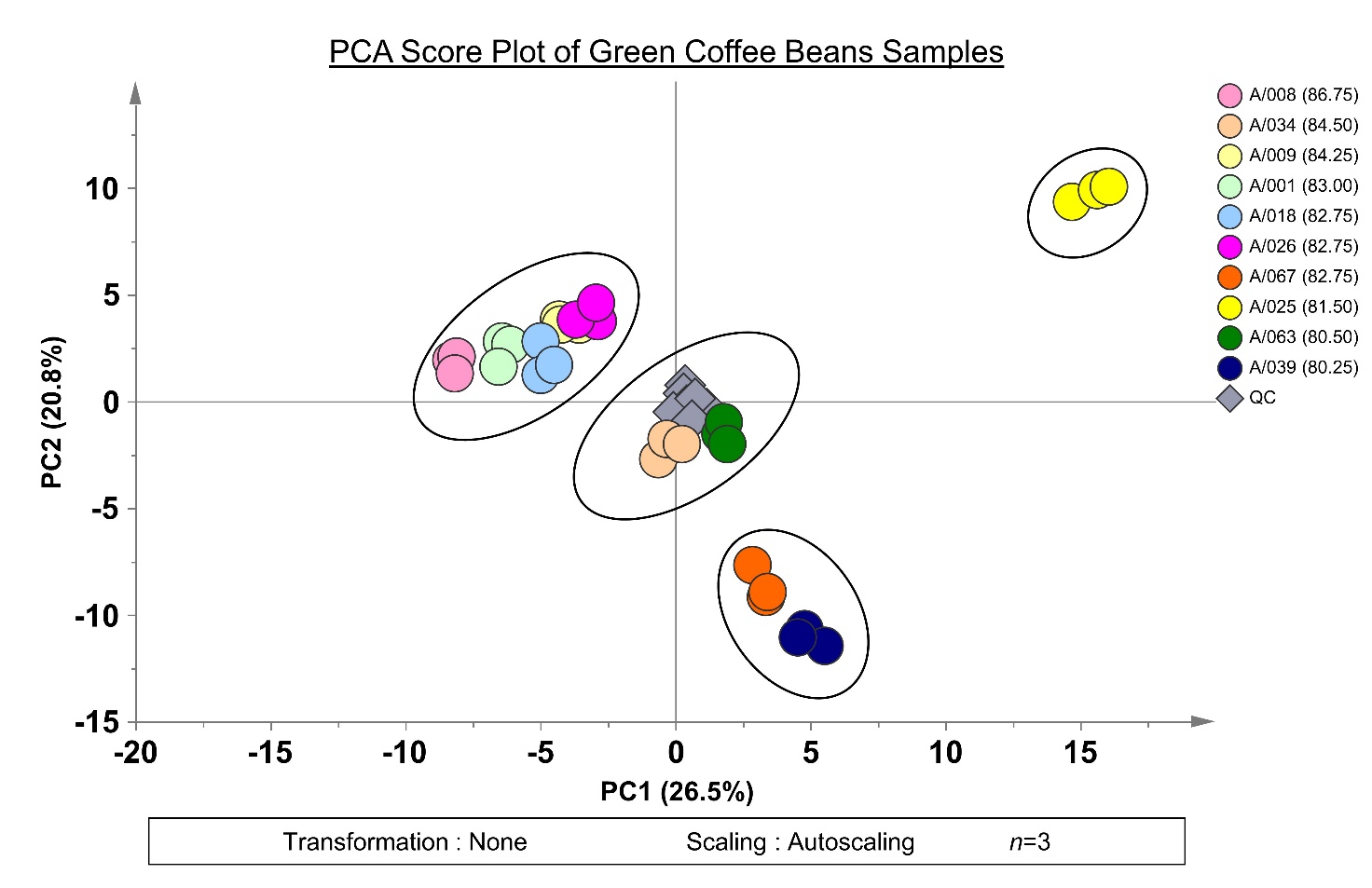


Figure S1. PCA score plot of 10 green coffee beans samples


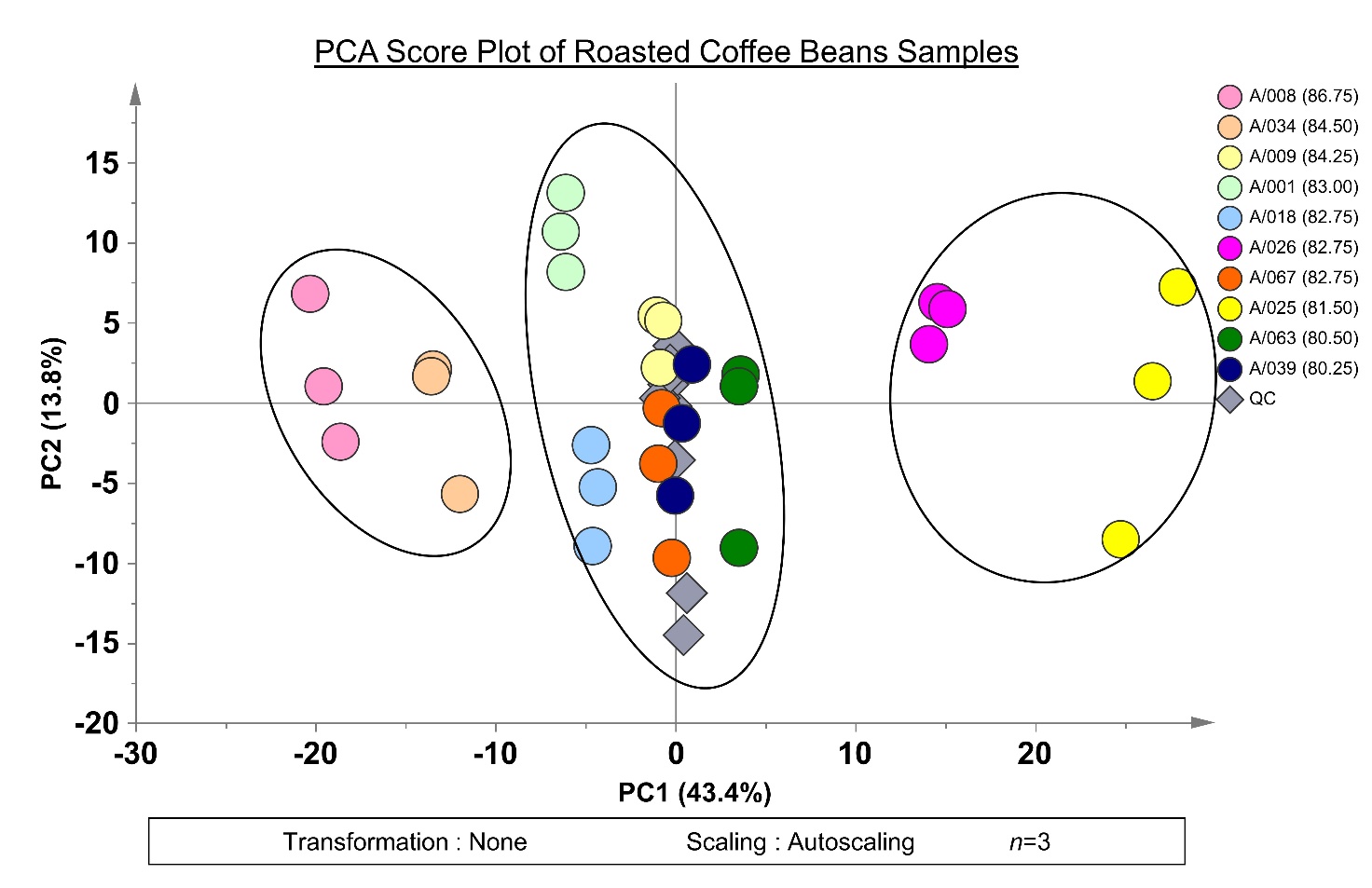


Figure S2. PCA score plot of 10 roasted coffee beans samples


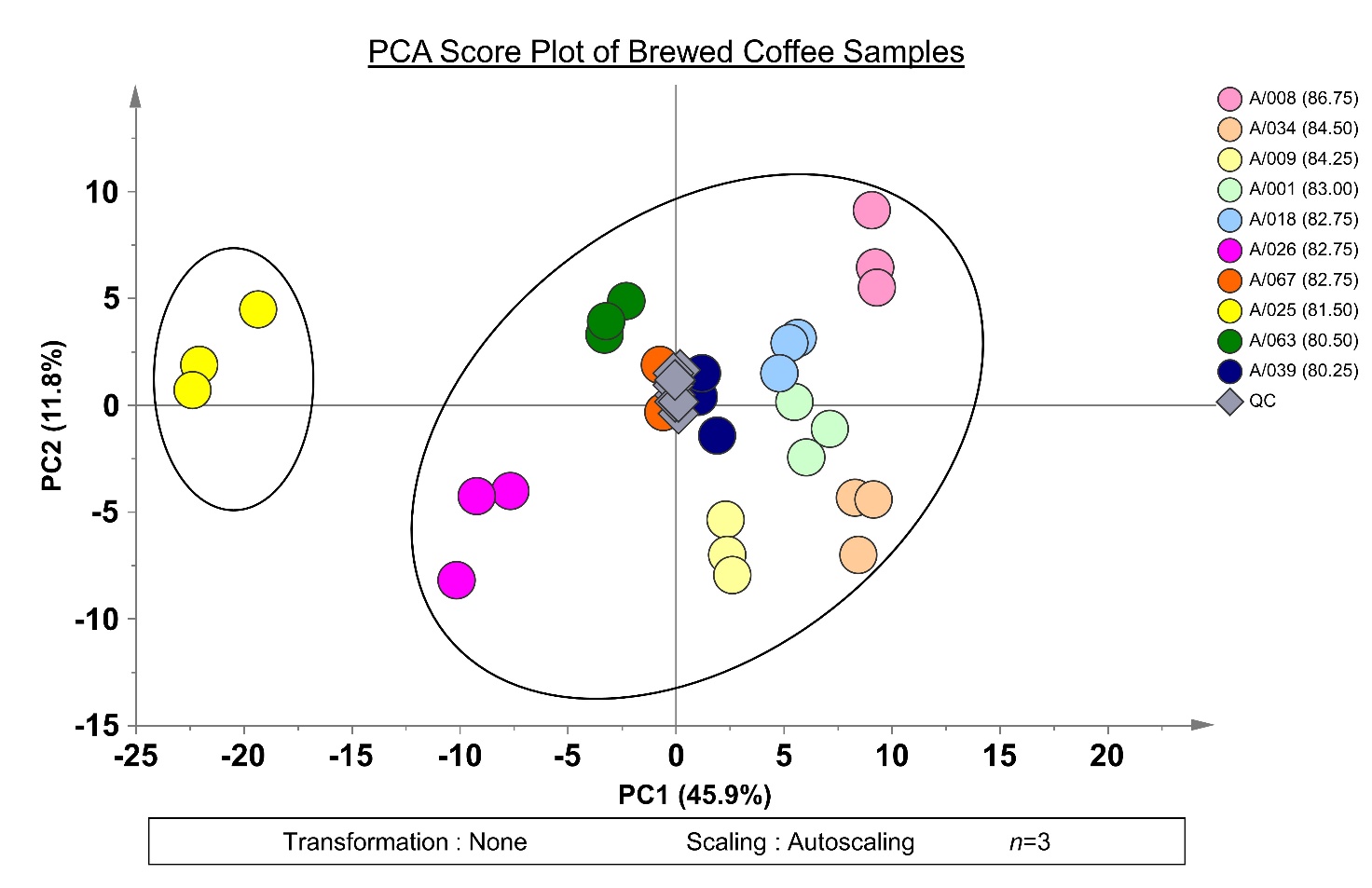


Figure S3. PCA score plot of 10 brewed coffee samples


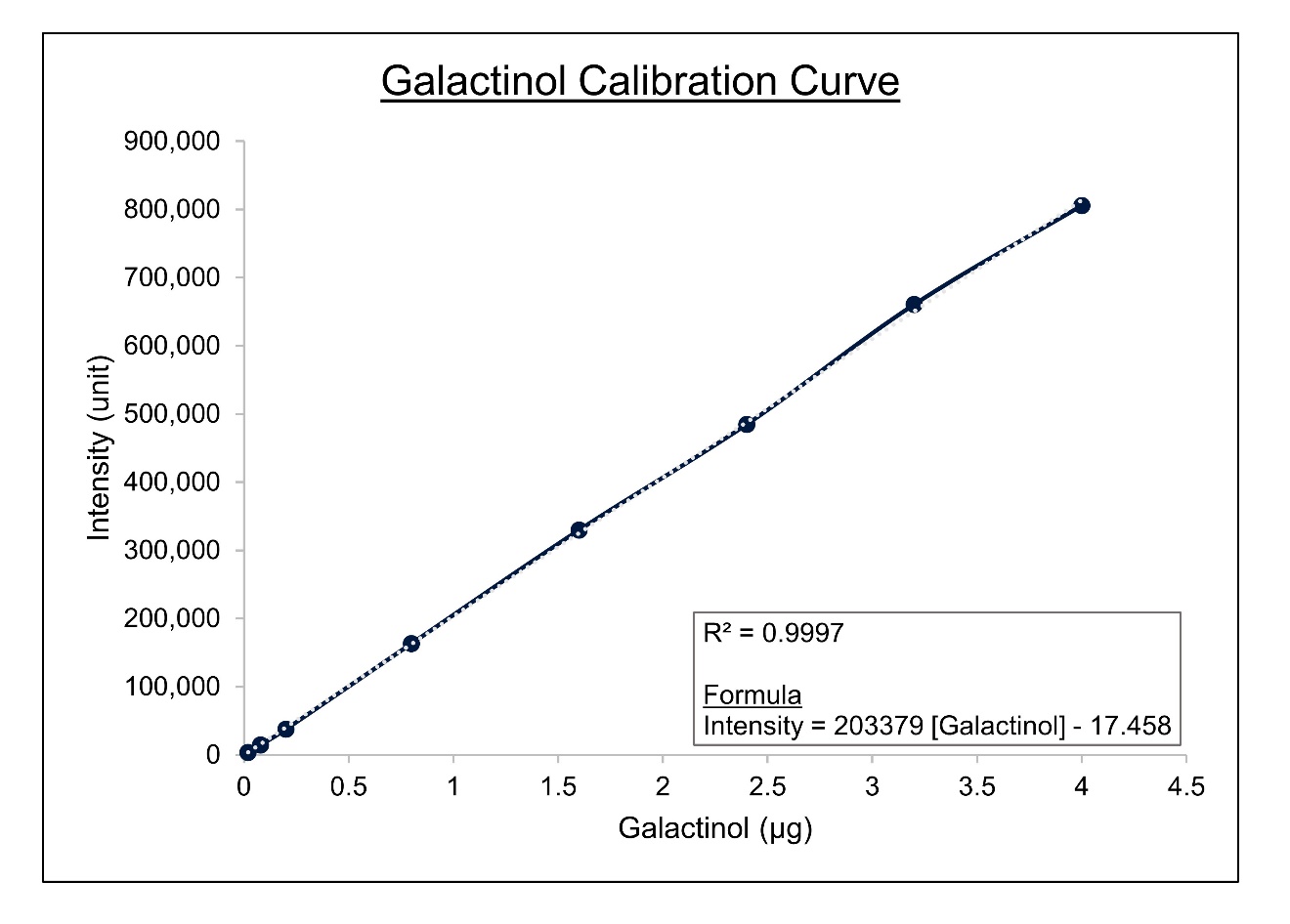


Figure S4. The galactinol calibration curve
